# Polymorph Selection and Derivatization in Enantiomerically Pure Medicarpin: Crystallographic and Computational Insights

**DOI:** 10.3390/molecules30173652

**Published:** 2025-09-08

**Authors:** Santiago José Guevara-Martínez, Rafael Herrera-Bucio, Marco Antonio Pérez-Cisneros, Gilberto Velázquez-Juárez, Fredy Geovannini Morales-Palacios, Stephanie García-Zavala

**Affiliations:** 1 Department of Pharmacology, School of Exact Sciences and Engineering, University of Guadalajara, Boulevard Gral. Marcelino García Barragán 1421, Olímpica, Guadalajara 44430, Jalisco, Mexico; santiago.guevara@academicos.udg.mx; 2 Instituto de Investigaciones Químico-Biológicas, Universidad Michoacana de San Nicolás de Hidalgo, Francisco J. Múgica, s/n, Morelia 58030, Michoacán, Mexico; rafael.herrera.bucio@umich.mx; 3 Department of Electrophotonics, School of Exact Sciences and Engineering, University of Guadalajara, Boulevard Gral. Marcelino García Barragán 1421, Olímpica, Guadalajara 44430, Jalisco, Mexico; marco.perez@cucei.udg.mx; 4 Department of Chemistry, School of Exact Sciences and Engineering, University of Guadalajara, Boulevard Gral. Marcelino García Barragán 1421, Olímpica, Guadalajara 44430, Jalisco, Mexico; gilberto.velazquez@academicos.udg.mx

**Keywords:** conformational polymorphism, chiral crystallography, isoflavonoid derivatives

## Abstract

Polymorphism critically influences the solid-state properties of organic molecules, affecting stability, solubility, and functionality. We investigated the polymorphic behavior of enantiomerically pure (+)-(6aS,11aS)-medicarpin through combined experimental and computational analyses. Single-crystal X-ray diffraction revealed two distinct chiral polymorphs: the previously reported monoclinic P2_1_ form and a newly identified orthorhombic P2_1_2_1_2_1_ form with a fully chiral packing arrangement. The discovery of this previously unreported polymorph underscores the subtle yet decisive effects of solvent and conformational flexibility in directing crystallization. Detailed structural analysis reveals that, whereas the P2_1_ form is only stabilized by a single dominant electrostatic interaction, the P2_1_2_1_2_1_ form features a more complex network comprising C-H···π contacts, bifurcated C-H···O hydrogen bonds, and aromatic edge-to-face interactions. Further investigation of a functionalized *p*-nitrobenzoate derivative corroborates the critical influence of molecular substituents and crystallization conditions on packing motifs. Lattice energy DFT calculations confirm that each polymorph is stabilized by distinct electrostatic and dispersive interaction patterns, illustrating the complex energetic landscape of polymorph selection. Altogether, this work provides a framework for understanding and anticipating which polymorph is likely to form under specific solvent and crystallization conditions, offering insights for future strategies in materials design and guiding the pursuit of patentable crystalline forms in pharmaceutical applications.

## 1. Introduction

Chiral polymorphism―the ability of an enantiomerically pure compound to crystallize in more than one distinct solid-state form―remains a central challenge in solid-state chemistry due to its impact on critical physicochemical properties such as stability, solubility, mechanical robustness, dissolution rate, and crystal morphology, all of which ultimately influence bioavailability and ADME profiles [1,2]. These variations originate from the delicate balance of noncovalent interactions within the crystal lattice, where subtle changes in molecular conformation or functionalization can lead to entirely different packing arrangements [3]. Recent methodological advances, combining high-resolution diffraction techniques with computational modeling and energy analysis, have expanded the range of known chiral polymorphs, yet conformational polymorphism―where intramolecular geometry changes produce alternative packing motifs―remains particularly difficult to predict. Even van der Waals-inclusive density functional theory (DFT) methods, despite many successes in crystal structure prediction (CSP), have shown systematic failures in correctly ranking the stability of such forms, due to inaccuracies in both intramolecular conformational energies and the descriptions of intermolecular interactions [4,5,6]. This persistent gap highlights the need for experimentally grounded studies on stereochemically rich molecules. 

Medicarpin (**1**), a bioactive isoflavonoid produced by leguminous species such as *Dalbergia*, *Erythrina*, and *Medicago* [7], has more recently been studied in callus and cell suspensions cultures [8], and through heterologous biosynthesis in microbial systems [9]. As a phytoalexin, it exhibits notable antimicrobial and antifungal activities [10,11,12,13], alongside antioxidant [14], anti-inflammatory [15], cardiovascular [16], and neuroprotective [17] properties, with emerging potential in bone regeneration, cancer therapy, and immune modulation [18]. While its pharmacological profile is diverse, medicarpin remains in the preclinical stage and, like many drug candidates, is expected to be formulated and administered in a crystalline form. Understanding its solid-state behavior is therefore strategically important, as unforeseen polymorphic transitions can alter performance, manufacturing reproducibility, and regulatory compliance―challenges exemplified by well-documented cases such as ritonavir and rotigotine [19]. Despite this, no comprehensive crystallographic or computational studies have been reported for medicarpin in the solid state; existing computational work has been limited to molecular docking studies, leaving its polymorphic landscape completely unexplored.

Notably, the monoclinic P2_1_ crystal form of medicarpin, denoted here as **1**(**I**), has been the only reported solid-state structure for over a decade. We report the discovery and full characterization of a second polymorph, **1**(**II**), crystallizing in the orthorhombic space group P2_1_2_1_2_1_, distinguished by a different conformation of the 3,4-dihydro-2H-pyran ring and a fully chiral packing arrangement. The new polymorph offers a compelling alternative: its higher symmetry and altered conformation suggest changes in packing efficiency and hydrogen-bonding networks, with possible enhancements in biopharmaceutical performance. Moreover, its isolation fills a long-standing structural gap and creates opportunities for solid-state optimization and intellectual property claims within drug development efforts.

To further probe the influence of minimal structural modifications, we synthesized the *p*-nitrobenzoate **2** derivative, which also crystallizes in P2_1_2_1_2_1_ and mirrors the fully chiral packing observed in **1**(**II**). Comparative analysis of these structures, supported by Hirshfeld surface and fingerprint mapping and energy framework calculations, reveals the strong role of intermolecular interactions and crystallization conditions in guiding early-stage solid-form outcomes [20,21,22]. Importantly, the computational analysis was divided into two complementary parts: single-point DFT energy calculations were performed to assess the conformational preferences of medicarpin at ambient temperature in an apolar solvent environment, while lattice energy decomposition using CrystalExplorer quantified intermolecular interaction contributions [23]. This dual strategy offers a comprehensive energetic perspective on polymorph stability and packing efficiency. Moreover, the present work includes the chiral HPLC identification of medicarpin obtained from a natural source, highlighting the critical importance of enantiomeric separation to isolate the single enantiomer (+)-(6a*S*,11a*S*)-medicarpin, as chiral purity directly influences polymorphic behavior. Overall, the significance of this study lies not in novel supramolecular description methods or energetic contributions, but in the discovery and detailed characterization of a new chiral polymorph of medicarpin, reflecting a broader trend in identifying rare and functionally important chiral polymorphs in natural products, as highlighted in recent reviews on enantiomeric distribution and characterization in nature [24].

## 2. Results and Discussion

### 2.1. Natural Extraction of Medicarpin and Synthesis of Derivatives

**Scheme 1:**
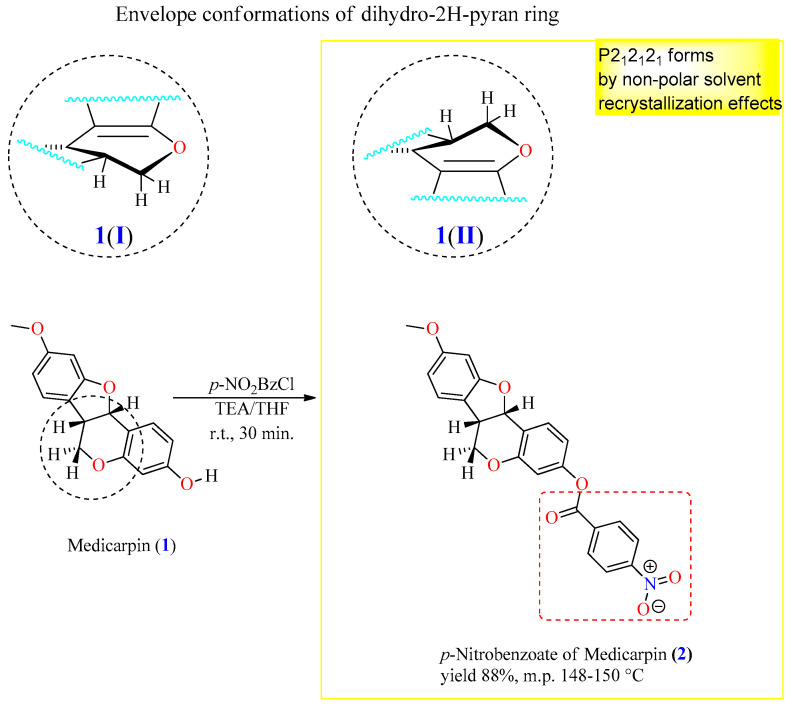
Conformational polymorphs of pyran ring (**I**) and (**II**) in (+)-(6a*S*,11a*S*)-medicarpin **1**, reaction conditions for derivative **2**.

The present study explores a molecular design approach centered on the conformational behavior of the 3,4-dihydro-2H-pyran ring in medicarpin (**1**) as a model system. To investigate the influence of targeted functionalization on crystal packing and supramolecular interactions, we began by isolating the parent compound from *Dalbergia congestiflora* Pittier using Soxhlet extraction. Crude extracts were obtained sequentially with hexane (*n*-hex) and ethyl acetate (EtOAc) over a period of 4 h. The EtOAc fraction was subsequently purified by column chromatography using a *n*-hex/EtOAc 97:3 solvent system, yielding pure (+)-(6a*S*,11a*S*)-medicarpin (**1**) (see Scheme 1) as a white solid. 

**Scheme 2:**
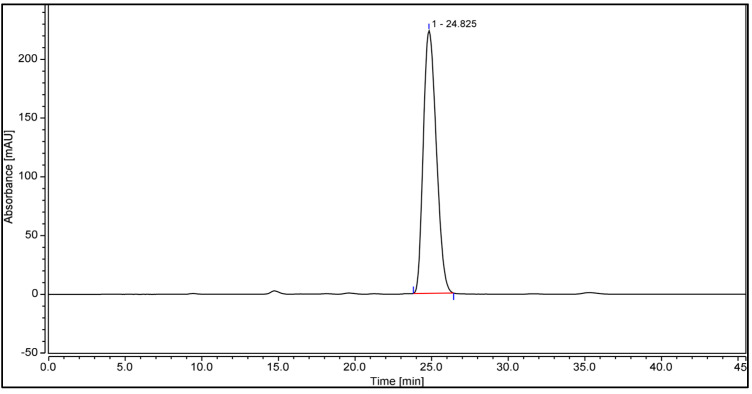
Chiral HPLC chromatogram confirming the enantiopurity of (+)-(6a*S*,11a*S*)-medicarpin indicating the exclusive presence of one enantiomer.

Enantiomeric purity was confirmed by HPLC analysis (method developed in this work, Scheme 2) using a CHIRALPAK AD-H (4.6 × 150 mm, 5 µm) column, with an *n*-hexane/isopropanol (90:10, *v*/*v*) mobile phase at 1.0 mL min^−1^, ambient temperature, and UV detection at 254 nm. The chromatogram displayed a single sharp peak at tR = 24.825 min, with an area of 220.407 mAU·min, height of 223.598 mAU, and relative area and height of 100%, confirming the exclusive presence of one enantiomer within the detection limits of the method. Prior studies have reported HPLC-based identification of medicarpin―for instance, in urine analysis following herbal medicine administration―and investigations of pterocarpan biosynthesis or metabolism using HPLC-based methods. However, these reports do not provide chromatographic details such as retention times or explicit HPLC conditions for medicarpin [25]. While chiral HPLC has been applied to resolve synthetic racemic pterocarpans, often with circular dichroism (CD), comprehensive chromatographic parameters for medicarpin remain scarce in the literature [26]. Polysaccharide-based chiral stationary phases such as those in the CHIRALPAK family are broadly employed for enantiomer separations across various natural product classes [27]. Thus, to the best of our knowledge, the current method represents the first detailed report of chiral HPLC separation of (+)-(6aS,11aS)-medicarpin using an AD-H column, providing a reproducible and effective protocol under well-defined chiral conditions.

To generate the *p*-nitrobenzoate derivative (**2**), compound **1** was subjected to esterification with *p*-nitrobenzoyl chloride in a TEA/THF (1:2 *v*/*v*) system, following a Schotten–Bauman protocol adapted from the literature [28,29]. Triethylamine was used as both base and co-solvent to neutralize the hydrochloric acid (HCl) released during the esterification process. Carrying out the reaction at room temperature (22–25 °C) helped minimize undesired side reactions and maintained clean conversion. The acylation could be conveniently monitored in real-time, via thin-layer chromatography (TLC), and afforded moderate to good yields under mild conditions. The resulting derivative **2** was purified and recrystallized from a *n*-hex/EtOAc (9:1) mixture, yielding single crystals suitable for X-ray diffraction. Both compounds, **1**(**II**) and **2**, were thoroughly characterized by spectral and crystallographic methods (see Supplementary Materials), enabling an integrated evaluation of structure-packing relationships relevant to rational solid-state design.

### 2.2. Crystallographic Characterization of Medicarpin Polymorphs *1(I)* and *1(II)*

**Figure 1:**
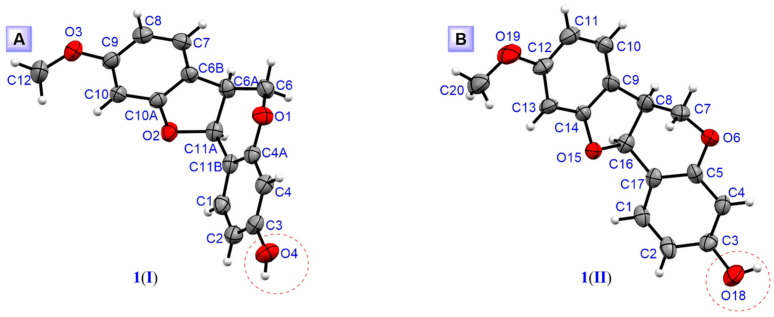
Three-dimensional ORTEP diagram of (+)-medicarpin (**1**): (**A**) monoclinic polymorph (**I**) in the P2_1_ space group, (with stereochemical centers 6a*S* and 11a*S*) and (**B**) orthorhombic polymorph **1**(**II**) in the P2_1_2_1_2_1_ space group (with stereochemical centers 8*S* and 16*S*). Atom labeling follows corresponding CIF entries. A red dotted circle highlights the hydroxyl position for subsequent derivatization.

The conformational polymorphism of enantiomerically pure (+)-(6a*S*,11a*S*)-medicarpin (**1**), C_16_H_14_O_4_, was investigated through single-crystal X-ray diffraction analysis (Figure 1), revealing for the first time the coexistence of two distinct enantiopure crystal lattices of the same natural product. Conformational analysis identified (A) the previously known monoclinic polymorph **1**(**I**) in the chiral space group P2_1_ (CCDC 750644) [3], and (B) the newly discovered orthorhombic polymorph **1**(**II**) in the chiral space group P2_1_2_1_2_1_ (CCDC 2405451). Differences in packing are attributed primarily to conformational flexibility of the 3,4-dihydro-2H-pyran ring. The isolation of **1**(**II**) is particularly impactful, as it demonstrates how a single chiral scaffold can adopt fundamentally different lattice arrangements under controlled crystallization. This behavior mirrors examples in materials chemistry―such as helicenes―where different chiral conformations lead to polymorphic control over properties such as circularly polarized luminescence and chiral charge transport [30,31]. In our case, this advantage is even more striking, as it depends solely on the conformational freedom of a single carbon atom in the rigid skeleton―C6 in **1**(**I**) or C7 in **1**(**II**)―underscoring a rare and precise control point for polymorphic engineering. While the ^1^H and ^13^C NMR spectra confirm the molecular structure (see Supporting Information), X-ray diffraction (XRD) analyses verify the solid-state form, further corroborating that the product obtained is the sole compound detected by HPLC.

The successful isolation of **1**(**II**) is particularly significant, as its higher symmetry and enhanced supramolecular network suggest increased thermodynamic robustness and potential utility in pharmaceutical or materials applications. Interestingly, polymorph **1**(**II**) displays a higher melting point (126–128 °C) than **1**(**I**) (117–118 °C), which correlates with the presence of a denser and more directional network of intermolecular noncovalent interactions. Moreover, although **1**(**II**) possesses a slightly lower calculated crystal density, the spatial arrangement of stabilizing interactions leads to greater packing efficiency compared with **1**(**I**), underscoring the non-trivial relationship between density, symmetry, and packing stability.

**Figure 2:**
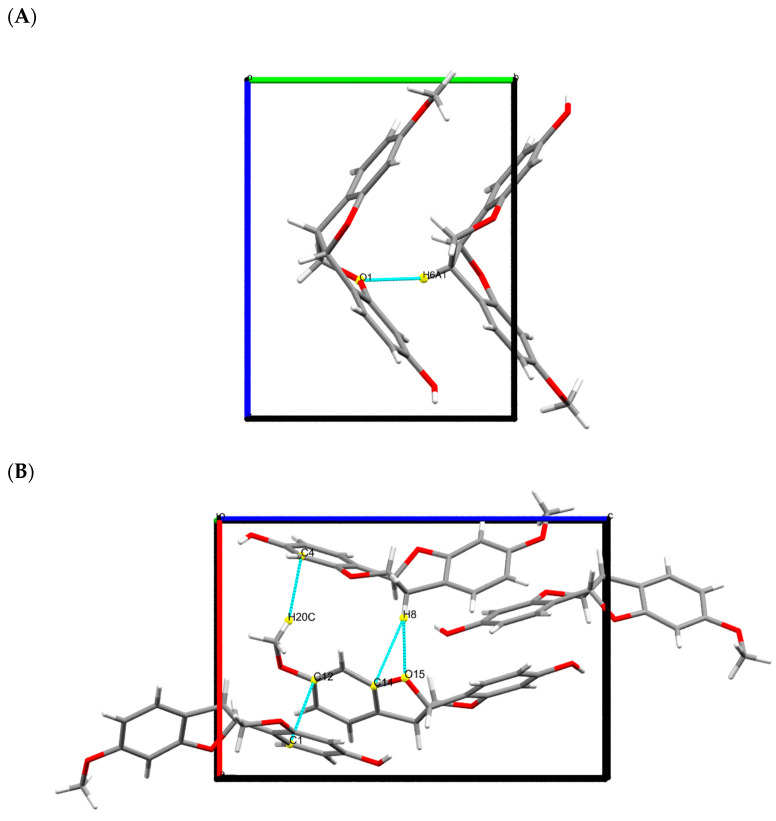
Crystal packing of **1**(**I**) visualized along the *a*-axis (**A**) and of **1**(**II**) along the *b*-axis (**B**).

A comparative packing analysis of both polymorphs reveals marked differences in their asymmetric units and supramolecular assemblies. In the monoclinic **1**(**I**) structure, stabilization is primarily mediated by a single electrostatic C-H···O contact between the pyran oxygen atom O1 and hydrogen H6A on an adjacent molecule (O1···H6A-C6A, 2.598 Å) (Figure 2A). This value is slightly shorter than the van der Waals limit for C-H···O contacts (~2.7 Å), placing it in the range of moderate hydrogen bonds, which―although individually weak―can provide relevant stabilization when acting cooperatively in the solid state [32].

In contrast, polymorph **1**(**II**) exhibits a more extensive three-dimensional hydrogen-bonding network, arising from three distinct supramolecular synthons: C-H···π interactions (C20-H20···C4, 2.896 Å), π-π interaction (C12···C1, 3.366 Å), and bifurcated C-H···O hydrogen bonds involving donor C8-H8 and acceptors C14 (C8-H8···C14, 2.893 Å) and O15 (C8-H8···O15, 2.646 Å) (Figure 2B). The C-H···π distance falls well within the established range (2.80–2.89 Å) for such interactions in crystal engineering, while the bifurcated C-H···O bonds reinforce lattice cohesion by both minimizing repulsive contacts and increasing packing efficiency. The π-π interaction is considered an aromatic edge-to-face (T-shaped) interaction between ring A of one medicarpin molecule and ring C of the adjacent molecule, with centroid (A)–centroid (C) of 4.192 Å, an interplanar angle of 89.66°, and a shortest edge-H···centroid distance that corresponds to 3.366 Å. The near-perpendicular orientation precludes significant π-π overlap; instead, stabilization arises from electrostatic attraction between the C-H bonds on the edge of ring A and the π-electron cloud of ring C, contributing to the greater structural rigidity of **1**(**II**).

Overall, these differences highlight the transition from a phase stabilized by a single moderate hydrogen bond in **1**(**I**) to a multi-interaction, three-dimensional network in **1**(**II**), a change that directly impacts crystal stability and conformational locking.

Solvent conditions played a decisive role in determining the polymorph outcome of (+)-medicarpin. Crystallization from a low-polarity *n*-hex/EtOAc (9:1 *v*/*v*) mixture consistently yielded the orthorhombic form **1**(**II**), a result that can be rationalized by reduced solvation of polar sites and an increased tendency for direct intermolecular recognition during nucleation. Conversely, recrystallization from more polar solvent systems, such as acetone/water [3], favored the monoclinic form **1**(**I**), likely because stronger solute–solvent interactions stabilize molecular conformations that are more compatible with its packing arrangement.

This solvent-driven selectivity is consistent with classical nucleation theory, which predicts that the preferential stabilization of certain conformations or pre-nucleation aggregates by the solvent can modify the relative free energy landscape, thereby altering the accessibility of competing polymorphs [33,34]. 

Even though the difference in dihedral angles between polymorphs **1**(**I**) and **1**(**II**) (shown in Table 1) is minuscule (~0.43°), this subtle shift represents a full conformational inversion (ring flip) of the pyran ring. Similarly, in highly polymorphic systems such as ROY derivatives, even torsion angle differences as small as 0.1° to 3.3° are sufficient to produce distinct crystal forms and colors [35]. These conformational inversion events are well documented in six-membered heterocycles such as tetrahydropyran, where small torsional adjustments are sufficient to cross energy barriers and shift from one puckered form to another. A detailed static and dynamical DFT study demonstrates that saturated six-membered rings undergo inversion transitions, passing through distinct conformational states such as chair, boat, and skew-boat, along well-defined pathways and energy barriers [36]. Given the extensive prior studies on heterocyclic ring inversion, a detailed DFT study of the inversion pathway is not required here; instead, we focus on comparing the relative energies of the two polymorphs, providing meaningful insight into their stability and predicted distribution in solution.

**Figure 3:**
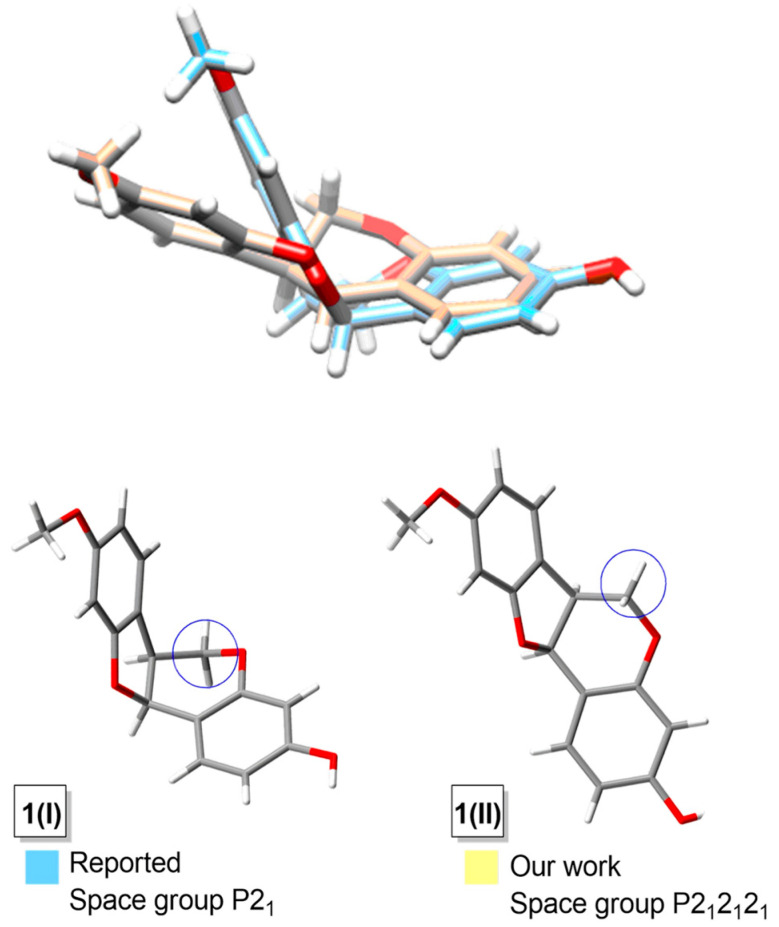
Overlay and conformational comparison between polymorphs **1**(**I**) and **1**(**II**).

Thermodynamic analysis using DFT single-point calculations (B3LYP/6-311++G(d,p)) on CIF-derived structures (Figure 3) revealed a negligible energy difference between **1**(**I**) (Δ*E* = −919.011584713 Ha) and **1**(**II**) (Δ*E* = −919.011668082 Ha) (Δ*E* ≈ −0.22 kJ/mol or −0.05258 kcal/mol). This indicates that both polymorphs occupy the same thermodynamic basin under ambient conditions. Therefore, polymorph selection appears to be dominated by solvent-mediated stabilization of specific conformations and nucleation pathways [37,38,39] rather than by intrinsic energetic preference.

The conformational divergence arises from torsional flexibility around the saturated C7 carbon within the 3,4-dihydro-2H-pyran ring, allowing the adoption of distinct envelope conformations. Solvent molecules present during nucleation can further influence hydrogen bonding, π-π aromatic edge-to-face (T-shaped) interaction, and CH-π interactions, directing the self-assembly toward one polymorph or the other [40].

### 2.3. Crystallographic Characterization of Medicarpin Polymorphs and Derivative *2*

The crystal structures of medicarpin and its derivatives reveal how subtle conformational differences and the presence of specific functional groups dictate packing preferences and polymorph formation. Medicarpin polymorphs **1**(**I**) and **1**(**II**) crystallize in the monoclinic (P2_1_) and orthorhombic (P2_1_2_1_2_1_) space groups, respectively, while derivative **2** (CCDC 2407462), functionalized with a bulky p-nitrobenzoate group, adopts the same orthorhombic symmetry after recrystallization under identical solvent conditions (*n*-hex/EtOAc 9:1) (Table 2). This phenomenon suggests that similar nucleation dynamics may be involved in the formation of both polymorphs. These structural variations underscore the influence of intermolecular interactions—particularly hydrogen bonding, dispersion, and steric effects—on molecular packing, in agreement with previous studies on polymorphism in organic compounds [41].

Regarding unit cell metrics, **1**(**I**) displayed the smallest volume (650.76 Å^3^, *Z* = 2), consistent with dense molecular packing and limited solvent-accessible voids. In contrast, the orthorhombic **1**(**II**) exhibited a significantly larger unit cell (1423.60 Å^3^, *Z* = 4), indicative of a more expanded hydrogen-bond network. Derivative **2**, bearing a bulky *p*-nitrobenzoate substituent, showed the largest cell volume (1928.6 Å^3^, *Z* = 4). These results are consistent with reports demonstrating that the introduction of sterically demanding groups can increase unit cell volume and crystal packing complexity [42]. 

Crystallographic refinement parameters provide additional insight into crystal quality and structural complexity. Polymorph **1**(**I**) yielded the lowest R_int_ value (0.013), indicating high-quality data and minimal scattering noise. By comparison, polymorph **1**(**II**) and derivative **2** exhibited moderately higher R_int_ values (0.020 and 0.029, respectively), reflecting increased structural complexity and greater challenges during refinement [43].

### 2.4. Analysis of the Supramolecular Motifs of Medicarpin Polymorphs and Derivative *2*

Building upon the previously discussed pyran ring conformational differences and solvent-mediated polymorph selection, supramolecular analysis (Table 3) reveals that polymorph **1**(**II**) exhibits a densely interconnected three-dimensional network, sustained by strong C-H···O hydrogen bonds, along with C-H···π, and π-π aromatic edge-to-face interactions. These interactions reinforce lattice cohesion, contributing to high crystal density, enhanced thermal stability, and increased mechanical resistance, while polar contacts may modulate solubility behavior. In contrast, derivative **2** is primarily stabilized by weaker interactions, such as C-H···O and C-H···π contacts, resulting in a more loosely packed, layered structure with reduced thermal stability and higher propensity for solvation in organic solvents.

The hydrogen-bonding network in **1**(**II**) adopts a ribbon-like motif, featuring bifurcated C-H···O and C-H···C interactions $\left(R_{1}^{2}\;(3\right)$ synthons D and E, respectively), which link molecules in a head-to-tail arrangement. These ribbons are further reinforced by secondary synthons (C-H···π, B, and π-π edge-to-face interactions, C), establishing a tightly interwoven 3D network that aligns with the high-density, high-stability character of this polymorph. By contrast, derivative **2** forms stacked molecular layers interconnected through C-H···π interactions (synthon F) and dipolar O=C···O-NO contacts (synthon G), the latter involving the ester carbonyl as donor and the nitro oxygen as an acceptor. This layered architecture promotes anisotropic crystal growth, reduces steric constraints, and enhances adaptability to solvation.

These supramolecular arrangements correlate with the previously observed differences in pyran ring conformations and the negligible energy differences from DFT lattice calculations, demonstrating how small torsional adjustments and functionalization guide self-assembly. The rigid 3D network of **1**(**II**) renders it suitable for applications in functional solid-state materials, such as heterogeneous catalysts [44], controlled drug-release matrices [45], and thermally stable optoelectronic devices [46]. In contrast, the more flexible, layered structure of **2** lends itself to anisotropic applications such as organic conductors, photonic crystals, or supramolecular assemblies [47]. Overall, these findings illustrate how molecular functionalization and subtle conformational preferences modulate crystalline architectures and physicochemical properties, providing a rational framework for the design of pharmaceutical and functional crystalline materials.

### 2.5. Hirshfeld Surface Analysis of (+)-(6aS,11aS)-Medicarpin Polymorphs and Derivative *2*

**Figure 4:**
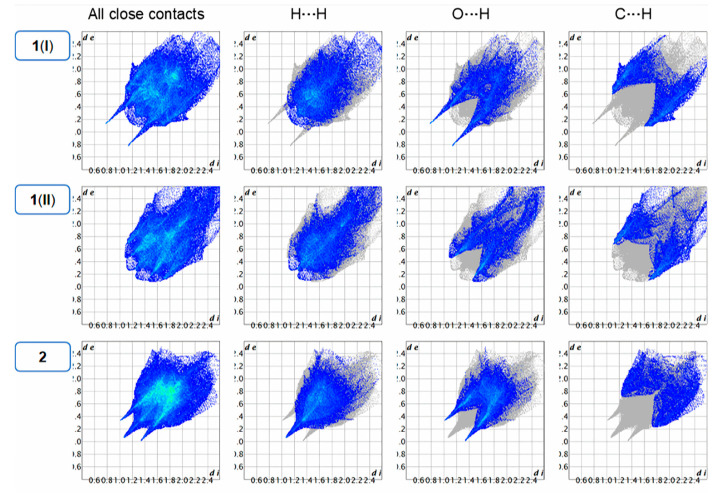
Hirshfeld two-dimensional fingerprint plots for each variation of intermolecular interactions among the polymorphs **1**(**I**–**II**) and derivative **2**.

Hirshfeld surface analysis (HSA) was conducted to complement the supramolecular characterization [20,21,22], with qualitative surfaces presented in the Supplementary Materials and quantitative insights derived from 2D fingerprint plots (Figure 4). The qualitative surfaces visually corroborate the key intermolecular synthons identified in Section 2.4, while the fingerprint plots provide a rigorous numerical assessment of short contacts shaping the crystal packing of both polymorphs of **1** and derivative **2**.

For all structures, H···H contacts dominate the Hirshfeld surface (44.1% in **1**(**I**), 46.9% in **1**(**II**), and 36.4% in derivative **2**) appearing as the central, dense regions of the fingerprint plots. O···H/H···O interactions rank second (27.3%, 26.0%, and 32.4%, respectively), followed by C···H/H···C contacts (25.5%, 23.5%, and 15.6%). In derivative **2**, a sharp H···H spike indicates a higher proportion of ordered short contacts, whereas in **1**(**I**) and **1**(**II**) these spikes are broader, reflecting greater variability in contact distances and weaker directionality.

Notable differences are also observed in the O···H/H···O interactions: **1**(**II**) displays broader, less defined spikes, whereas **1**(**I**) exhibits sharper and more distinct peaks. Upon derivatization, compound **2**, adopting the same conformation as **1**(**II**), restores the lost definition and even enhances the strength of these interactions. C···H/H···C contacts—often visualized as the “wings” in fingerprint plots [48]—are most pronounced in **1**(**I**) (25.5%), slightly reduced in **1**(**II**) (23.5%), and minimal in derivative **2** (15.6%), consistent with more ordered hydrophobic interactions in the monoclinic form.

Overall, the combined HSA and fingerprint analysis quantitatively reinforces the supramolecular trends discussed in Section 2.4, highlighting how conformational variation and functional group modification modulate both the prevalence and the geometry of key intermolecular contacts in these systems.

### 2.6. Theoretical Calculation of Crystal Lattice Energy Contributions

The crystal lattice interaction energies for the studied polymorphs were decomposed into their four principal components―electrostatic (*E_Cou_*), dispersion (*E_disp_*), repulsion (*E_rep_*), and polarization (*E_pol_*)―following the energy framework methodology implemented in CrystalExplorer [23]. The pairwise interaction energies were computed using the B3LYP/6-31G(d,p) level theory, and the unscaled total energy for each molecular pair was expressed as

*E_tot_* = *E_ele_* + *E_pol_* + *E_dis_* + *E_rep_* (1)

where each term corresponds, respectively to Coulombic (electrostatic) interactions between charge distributions, the distortion of the electron cloud induced by neighboring charges, the attractive van der Waals dispersion forces, and the short-range exchange-repulsion preventing molecular overlap. In accordance with Mackenzie et al. [49], the total energies reported here are the sum of these four energy components, each scaled appropriately by the scale factor for the energy model used (Equation (2)), as summarized in Table 4:

*E_tot_* = *k_ele_ E′_ele_* + *k_pol_ E′_pol_* + *k_dis_ E′_dis_* + *k_rep_ E′_rep_* (2)

For polymorph **1**(**I**), the most significant intermolecular interaction occurs at a centroid–centroid distance of 11.32 Å, with *E_tot_* = −32.30 kcal/mol. This interaction profile is dominated by electrostatic attraction (*E_Cou_* = −33.40 kcal/mol) offset by substantial repulsion (*E_rep_* = 38.30 kcal/mol) consistent with a densely packed, mechanically rigid arrangement. Such a configuration implies limited structural flexibility, favoring applications where hardness and stability are critical, such as molecular sieves or charge-sensitive materials [50,51,52]. The electrostatically dominated architecture also correlates with crystallization from polar solvents, where Coulombic interactions are thermodynamically enhanced [53]. 

In contrast, polymorph **1**(**II**) attains its energetic minimum at a shorter centroid distance of 5.07 Å, showing a more negative total lattice energy (*E_tot_* = −34.50 kcal/mol). Here, dispersion forces dominate (*E_disp_* = −43.70 kcal/mol), while repulsion is markedly lower (*E_rep_* = 25.40 kcal/mol), indicating a less densely packed but more conformationally adaptable crystal network, potentially advantageous for dynamic host–guest systems or stimuli-responsive materials.

These features align with a supramolecular framework primarily stabilized by van der Waals interactions, imparting enhanced resistance to thermal and pressure variations [53,54]. Such stabilization renders polymorph **1**(**II**) an attractive candidate for layered materials and organic semiconductor applications [44,45,46]. Its experimental isolation under low-polarity crystallization conditions (*n*-hex/EtOAc, 9:1), is consistent with the dominance of dispersion forces, which are preferentially expressed in nonpolar media. Conversely, the crystallization of **1**(**I**) under more polar conditions reflects the stabilization of electrostatic contacts in solution, favoring nucleation toward the monoclinic form [54,55].

From a broader polymorph screening perspective, these findings emphasize that the most thermodynamically favored structure―in this case, **1**(**II**)―may not always prevail during crystallization. Subtle stereochemical perturbations during synthesis can introduce competing nucleation pathways, ultimately influencing the phase outcome. For materials design, **1**(**II**) remains the more robust and thermodynamically stable option [54,56,57], while **1**(**I**), although slightly less stable, is crystallographically accessible under polar conditions.

In contrast, compound **2** exhibits pronounced dispersion contributions (*E_disp_* = −55.80 kJ·mol^−1^ at 8.50 Å) complemented by moderate electrostatic terms, yielding total interaction energies *E_tot_* = −42.50 to −11.10 kJ·mol^−1^. This dispersion-dominated yet balanced packing within the orthorhombic lattice supports greater conformational adaptability, favoring reversible self-assembly and molecular recognition processes. 

Taken together, these energetic profiles illustrate the distinct packing preferences across the studied systems and set the ground for a broader reflection on the scope and limitations of the computational approach employed. In this context, while the energy framework analysis with *CrystalExplorer* is intrinsically semi-empirical and does not rigorously capture the collective effects of crystal periodicity, it provides a practical and widely adopted tool to compare the relative contributions of electrostatics, dispersion, repulsion, and polarization in molecular packing. We acknowledge that periodic DFT approaches (e.g., WIEN2k, Crystal) would offer a more rigorous description of electron density and lattice energy; however, such calculations fall outside the scope of the present study, which was specifically designed to emphasize the experimental elucidation of polymorph selection and derivatization and their correlation with supramolecular interaction trends. Within this framework, *CrystalExplorer* serves as a complementary computational descriptor that supports the crystallographic findings and highlights packing preferences, rather than as a comprehensive theoretical treatment of crystal stability.

## 3. Materials and Methods

The parent compound (+)-(6a*S*,11a*S*)-medicarpin (**1**) was isolated from the heartwood of *Dalbergia congestiflora* Pittier. The dried tree material was subjected to Soxhlet extraction sequentially with n-hexane (n-hex) and ethyl acetate (EtOAc) for a total duration of 4 h. The EtOAc extract was concentrated under reduced pressure and subsequently purified by column chromatography using a *n*-hex/EtOAc (97:3) solvent system as eluent, affording (+)-(6a*S*,11a*S*)-medicarpin (**1**) as a white solid, which was used without further modification for crystallographic and derivatization studies. The reaction used chemicals of analytical grade, sourced from Sigma Aldrich/Merck (St. Louis, MO, USA) and utilized without any additional purification. Hexane (*n*-hex) and ethyl acetate (EtOAc) were freshly distilled using a fractional Vigreux column. Pyridine, used both as a base and solvent, was the preferred choice for reactions involving derivatives and was employed directly for its analytical grade without further purification. Column chromatography was conducted using Merck Silica Gel (70–230 Mesh), while TLC analysis was performed on Merck 60-F25 plates, visualized under UV light. Melting points were measured on a Fischer model 1237 apparatus and are uncorrected. IR spectra were recorded with a Thermo Scientific Nicolet iS10 instrument (Waltham, MA, USA), and NMR spectra were acquired on a Varian Mercury Plus spectrometer (100 and 400 MHz) (Agilent, Santa Clara, CA, USA). Mass spectra were obtained using a Thermo Scientific ISQ CT spectrometer with electron impact ionization. 

### 3.1. FT-IR, NMR, and MS Spectroscopic Studies

ATR/FT-IR spectra were recorded within the 4000–600 cm^−1^ range on a Thermo Scientific Nicolet iS10 spectrometer. NMR spectra were acquired with a Varian Mercury plus spectrometer, operating at frequencies of 400 MHz for ^1^H and 100 MHz for ^13^C nuclei. Mass spectra were obtained using a Thermo Scientific ISQ CT spectrometer with electron impact ionization.

### 3.2. HPLC Analysis

The enantiomeric purity of (+)-(6aS,11aS)-medicarpin was determined by high-performance liquid chromatography (HPLC) using a CHIRALPAK^®^ AD-H column (4.6 × 150 mm, 5 µm; Daicel Chemical Industries, Ltd. Tokyo, Japan; Himeji, Hyogo, Japan). The mobile phase consisted of n-hexane/isopropanol (90:10, *v*/*v*) at a flow rate of 1.0 mL/min. Analyses were performed at ambient temperature with UV detection at 254 nm. Samples were dissolved in the mobile phase and injected in a volume of 20 µL. Chromatographic data (retention time, area, and peak height) were recorded and processed using the instrument’s integrated software.

### 3.3. X-Ray Diffraction Studies

Single crystals suitable for X-ray diffraction were grown at room temperature over a week through slow evaporation of a 9:1 *n*-hex/EtOAc mixture, then selected via optical microscopy. Crystallographic data for compounds **1**(**II**) and **2** were collected using an Oxford Diffraction Xcalibur S system with a Sapphire 3 CCD detector. Diffraction experiments were carried out at approximately 22–25 °C. Data integration and scaling were processed with the CrysAlis PRO [58] and APEX3 v2018.1-0 (Bruker AXS) software suites [59], and the structural models were refined with SHELXL-2019/2 software package through direct methods [60], full-matrix least-squares refinement and SIR-92. Non-hydrogen atoms were refined anisotropically, while hydrogen atoms were positioned in calculated idealized positions and refined as riding on their parent atoms using standard parameters. A detailed summary of data collection, refinement details, and other structural figures were produced by Mercury [61], Chimera [62], and CrystalExplorer 17 [22,23]. CIF files containing supplementary crystallographic data were deposited at Cambridge Crystallographic Data Centre with references CCDC 2405451 for polymorph **1**(**II**) and CCDC 2407462 for compound **2**.

### 3.4. Quantum Chemistry Studies

CIF files and structural parameters derived from single-crystal X-ray diffraction (XRD) of compounds **1**(**II**) and **2** were used for solid-state surface calculations mapped over the electrostatic potential using the computational chemistry package Tonto, integrated into the CrystalExplorer 17.5.0.20210711 program [23]. This enabled the quantification and visualization of intermolecular interaction donors and acceptors through Hirshfeld surface analysis, as well as the calculation of energy contributions relevant to crystal conformational analysis. The Tonto package employs B3LYP/DFT wavefunction calculations based on the input CIF files. Additionally, DFT was applied in single-point calculations for the conformers, using the B3LYP functional at the 6-311++G(*d*,*p*) basis set in the Gaussian 16 software suite [63].

### 3.5. Synthetic Procedures

#### 3.5.1. General Procedure for Synthesis of (6aS,11aS)-9-Methoxy-6a,11a-dihydro-6H-benzofuro [3,2-c]chromen-3-yl 4-nitrobenzoate (**2**)

Thirty milligrams of (+)-(6aS,11aS)-medicarpin was reacted with 0.3 mL of *p*-nitrobenzoyl chloride and 0.3 mL of TEA, using anhydrous THF as the solvent under nitrogen atmosphere, for 24 h at room temperature. After the reaction time had elapsed, the mixture was washed twice with 30 mL of EtOAc, followed by two washes with 50 mL of 10% hydrochloric acid (HCl) solution, two washes with 30 mL of saturated sodium bicarbonate (NaHCO_3_) solution, and two washes with 30 mL of saturated sodium chloride (NaCl) solution. The organic phase was then dried over anhydrous sodium sulfate, filtered, and the solvent was evaporated under reduced pressure using a rotary evaporator, yielding a yellow oil. The crude product was subsequently purified by column chromatography, employing silica gel as the stationary phase and *n*-hex/EtOAc (90:10) as the mobile phase. This process obtained yellow needle-shaped single crystals, identified as medicarpin *p*-nitrobenzoate.

#### 3.5.2. General Procedure for Synthesis of (6aS,11aS)-9-Methoxy-6a,11a-dihydro-6H-benzofuro [3,2-c]chromen-3-yl diethylcarbamate (**3**)

Thirty milligrams of (+)-(6aS,11aS)-medicarpin was reacted with 0.1 mL of diethylcarbamoyl chloride and 2 mL of pyridine, used both as base and solvent, under reflux conditions and nitrogen atmosphere for 3 h. After the reaction time had elapsed, the mixture was washed successively with EtOAc, a 10% HCl solution, a saturated NaHCO_3_ solution, and a saturated NaCl solution. The crude reaction mixture was purified by column chromatography, using silica gel as the stationary phase and n-hex/EtOAc (95:5) as the mobile phase. This process yielded white crystals, identified as medicarpin diethylcarbamate ester.

## 4. Conclusions

Our investigation demonstrates that polymorph selection in enantiomerically pure (+)-(6a*S*,11a*S*)-medicarpin (**1**) can be directed through solvent-controlled crystallization and strategic molecular derivatization. We report and thoroughly characterize two true chiral polymorphs of **1**―designated as **1**(**I**) and **1**(**II**)―with the latter representing a newly isolated orthorhombic P2_1_2_1_2_1_ form. Alongside these findings, we also describe compound **2**, a hydroxyl-functionalized derivative of **1**. While not a polymorph of **1**, its crystallization under comparable low-polarity conditions demonstrates how specific functionalization strategies can influence intermolecular forces and packing motifs without inducing racemization. Any investigation into the potential polymorphism of **2** lies beyond the present study.

The discovery and isolation of **1**(**II**) exemplifies the labile nature of polymorphic crystallization, where subtle solvent effects and restricted conformational flexibility at a single carbon can drastically shift packing preferences. Lattice energy calculations indicate that **1**(**II**) attains greater thermodynamic stability through strong dispersion interactions, in contrast to the electrostatically dominated, more rigid packing of **1**(**I**).

Overall, the integrated experimental–computational approach presented here provides a framework for crystal engineering that is predictive in the sense that it enables anticipation of which polymorph is likely to form under specific solvent and crystallization conditions. Additionally, the derivatization of natural scaffolds such as medicarpin offers a versatile strategy to influence specific intermolecular interactions. These insights may guide the future development of advanced materials in pharmaceuticals, optoelectronics, porous frameworks, and functional devices.

## Figures and Tables

**Table 1:** Structural conformation and dihedral angle of polymorphs **1**(**I**) and **1**(**II**).

Crystal Form	Bond	Dihedral Angle (*τ*)
** 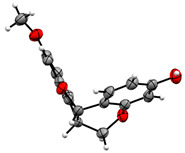 ** **1(I)**	C11A-C6A-C6-O1 ^[a]^	112.14°
** 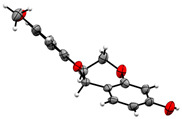 ** **1(II)**	C16-C8-C7-O6 ^[b]^	112.57°

^[a]^ Atom labels used in reported CIF CCDC 750644; ^[b]^ Atom labels used in reported CIF CCDC 2405451.

**Table 2:** Experimental crystallographic data of each medicarpin variation of **1** and derivative **2**.

Crystal Data	1(I)	1(II)	2
Chemical formula	C_16_H_14_O_4_	C_16_H_14_O_4_	C_23_H_17_NO_7_
*M*_r_	270.27	270.27	419.38
Crystal system	Monoclinic	Orthorhombic	Orthorhombic
Space group	*P*2_1_	*P*2_1_2_1_2_1_	*P*2_1_2_1_2_1_
Temperature (K)	293	292	293
*a*, *b*, *c* (Å)	6.6289 (3), 8.7963 (4), 11.3150 (5)	9.7932 (4), 9.8680 (7), 14.7311 (4)	6.6717 (6), 13.5236 (12), 21.3751 (17)
*Β*	99.482 (1)°	90°	90°
*V* (Å^3^)	650.76 (5)	1423.60 (12)	1928.6 (3)
*Z*	2	4	4
Radiation type	Mo *K*α	Mo *K*α	Mo *K*α
µ (mm^−1^)	0.10	0.09	0.11
Crystal size (mm)	0.40 × 0.25 × 0.20	1.01 × 0.67 × 0.42	0.48 × 0.46 × 0.35
Diffractometer	Bruker *SMART*	Oxford Diffraction Xcalibur S	Oxford Diffraction Xcalibur S
No. of measured, independent, and observed [*I* > *2σ*(*I*)] reflections	4783, 3198, 1949	7817, 3975, 3234	11,708, 6160, 3770
*R*_int_	0.013	0.020	0.029
(sin *θ*/*λ*)_max_ (Å^−1^)	0.712	0.755	0.758
**Refinement**	**1**(**I**)	**1**(**II**)	**2**
*R*[*F*^2^ > 2*σ*(*F*^2^)] *wR*(*F*^2^) *S*	0.034, 0.093, 1.09	0.169 0.457 1.92	0.061 0.121 1.09
No. of reflections	1949	3975	6160
No. of parameters	182	183	281

**Table 3:** Supramolecular synthons on close contact interactions for each polymorph **1**(**I**) and **1**(**II**) and derivative compound **2**.

Compound	Synthon	2D Diagram	3D Diagram
**1**(**I**)	(A) C-H···O	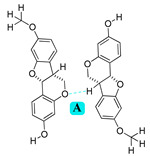	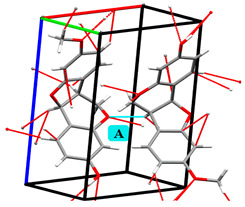
**1**(**II**)	(B) C20-H20···C4 (C) C1···C12 (D) C8-H8···C14 (E) C8-H8···O15	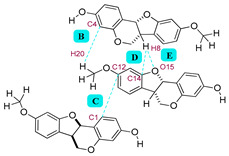	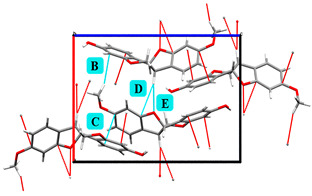
**2**	(F) C31-H31···C16 (G) C1···O11	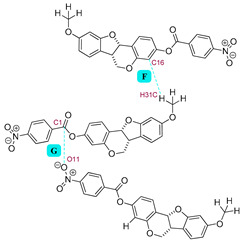	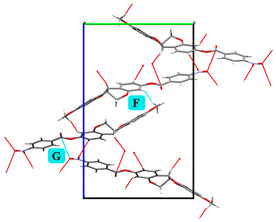

**Table 4:** Intermolecular interaction energy contributions (kJ mol^−1^).

	Crystal Structure			Energy Contributions (kJ mol^−1^)					
Polymorph **1**(**I**):	**1**(**I**)		Symmetry	Centroid	*E_Cou_*	*E_pol_*	*E_disp_*	*E_rep_*	*E_tot_*
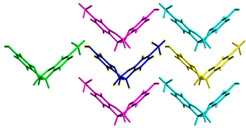			−*x*,*y* + 1/2, −*z*	5.1	−12.6	−3.6	−56	31.2	−45.4
			*x*,*y*,*z*	11.32	−33.4	−7.5	−17.4	38.3	−32.3
			*x*,*y*,*z*	12.14	−2.3	−0.6	−6.1	0	−8.1
			−*x*,*y* + 1/2, −*z*	11.36	−3.2	−0.7	−9	7.2	−7.3
			*x*,*y*,*z*	6.63	−8.7	−2.5	−26.5	20.1	−21.7
			−*x*,*y* + 1/2, −*z*	6.09	−10.1	−2.6	−34.2	21.6	−29
	Σ *E_tot_* = −143.8 kJ·mol^−1^								
Polymorph **1**(**II**):	**1**(**II**)		−*x* + 1/2, −*y*,*z* + 1/2	8.48	−5.7	−1.4	−35	21.8	−24.1
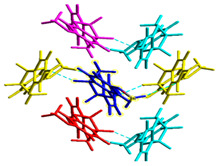			*x* + 1/2, −*y* + 1/2, −*z*	5.07	−9.8	−2.3	−43.7	25.4	−34.5
			−*x*, *y* + 1/2, −*z* + 1/2	8.92	−5.7	−1	−19.5	12.4	−16.1
			−*x*,*y* + 1/2, −*z* + 1/2	9.75	−1.9	−1	−16.7	7.5	−12.6
			*x*,*y*,*z*	14.72	−8.3	−0.7	−4.3	0	−13.1
			−*x* + 1/2, −*y*,*z* + 1/2	9.8	−0.1	−0.7	−9.6	4.1	−6.4
	Σ *E_tot_* = −106.8 kJ·mol^−1^								
Derivative **2**:	**2**		−*x*,*y* + 1/2, −*z* + 1/2	11.77	−6.7	−2	−10.8	8.5	−12.7
** 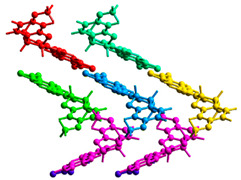 **			*x*,*y*,*z*	15.08	0.5	−1.8	−26	0	−23.4
			*x*,*y*,*z*	6.67	−7.7	−2.8	−35.9	16.6	−31.3
			−*x*,*y* + 1/2, −*z* + 1/2	8.75	−2.5	−1.6	−42.6	18.8	−29.2
			*x*,*y*,*z*	13.52	−4.8	−1.5	−24.6	0	−27.7
			*x* + 1/2, −*y* + 1/2, −*z*	8.5	−7.7	−2.4	−55.8	25.9	−42.5
			*x* + 1/2, −*y* + 1/2, −*z*	20.27	−4	−0.6	−7.4	0	−11.1
	Σ *E_tot_* = −177.9 kJ·mol^−1^								
	*k* constants=	*kE_Cou_* = 1.057	*kE_pol_* = 0.740	*kE_disp_* = 0.871		*kE_rep_* = 0.618			

## Data Availability

All data generated or analyzed during this study are included in this article and its Supplementary Materials Files. The Supplementary Material File encompasses comprehensive experimental details, including chemical characterizations (NMR, FT-IR, MS), full crystallographic images, and quantum chemical calculations. Crystallographic data for the structures reported in this work have been deposited in the Cambridge Crystallographic Data Centre (CCDC) under the deposition numbers 2405451, 2407462, and 2421041, and can be obtained free of charge via https://www.ccdc.cam.ac.uk/data_request/cif (accessed on 29 August 2025).

## Supplementary Materials

The following supporting information can be downloaded at: https://www.mdpi.com/article/10.3390/molecules30173652/s1, Figure S1: Full-size crystal packing image of compound **1(II)** with short contact interactions shown as dashed red lines, and blue ones for H-bonding. Visualization by symmetry elements on the *a*-axis. Figure S2: Hirshfeld surface image of compound **1(II)** with H-bonding shown as dashed blue lines. Visualization by on the *b*-plane. Figure S3: Energy framework dispersion diagram for a cluster of molecules of compound **1(II)**, on the *a*-plane. Figure S4: Energy framework dispersion diagram for a cluster of molecules of compound **1(II)**, on the *b*-plane. Figure S5: Full-size crystal packing image of derivative 2 with short contact interactions shown as dashed red lines, and blue ones for H-bonding. Visualization on the *b*-axis. Figure S6: Full-size crystal packing image of derivative 2 with short contact interactions shown as dashed red lines, and blue ones for H-bonding. Visualization by symmetry elements on the *b*-axis. Figure S7: Hirshfeld surface image of derivative 2 with H-bonding shown as dashed blue lines. Visualization by on the *a*-plane. Figure S8: Hirshfeld surface image of derivative 2 with H-bonding shown as dashed blue lines. Visualization by on the *b*-plane. Figure S9: Energy framework dispersion diagram for a cluster of molecules of compound **2**, on the *a*-plane. Figure S10: Energy framework dispersion diagram for a cluster of molecules of compound **2**, on the *b*-plane. Table S1: Atomic coordinates (×10^4^) and equivalent isotropic displacement parameters (Å^2^ × 10^3^) for **1(II)**. U(eq) is defined as one third of the trace of the orthogonalized U^ij^ tensor. Table S2: Bond lengths [Å] and angles [°] for **1(II)**. Table S3: Hydrogen bonds for **1(II)** [Å and °]. Table S4: Anisotropic displacement parameters (Å^2^ × 10^3^) for **1(II)**. The anisotropic displacement factor exponent takes the form: −2 ^2^[h^2^a*^2^U^11^ + … + 2 h k a* b* U^12^]. Table S5: Atomic coordinates (×10^4^) and equivalent isotropic displacement parameters (Å^2^ × 10^3^) for **2**. U(eq) is defined as one third of the trace of the orthogonalized U^ij^ tensor. Table S6: Bond lengths [Å] and angles [°] for **2**. Table S7: Hydrogen bonds for **2** [Å and °]. Table S8: Anisotropic displacement parameters (Å^2^ × 10^3^) for 2. The anisotropic displacement factor exponent takes the form: −2 ^2^[h^2^a*^2^U^11^ + … + 2 h k a* b* U^12^]. Table S9: Cartesian coordinates and energies optimized by B3LYP/6-311G(*d*,*p*) for **1(I)**. Table S10. Cartesian coordinates and energies optimized by B3LYP/6-311G(*d*,*p*) for **1(II)**.
