# Supplementary material for: Polymorph Selection and Derivatization in Enantiomerically Pure Medicarpin: Crystallographic and Computational Insights
Source: Molecules. 2025 Sep 8;30(17):3652. doi: 10.3390/molecules30173652 (PMC12430461; doi:10.3390/molecules30173652)
Supplement: Supplementary file 1 [file molecules-30-03652-s001.zip › molecules-3805445-supplementary.pdf]

Polymorph Selection and Derivatization in Enantiomerically Pure Medicarpin: Crystallographic and Computational Insights

Santiago José Guevara-Martínez 1, Rafael Herrera-Bucio 2, Marco Antonio Pérez-Cisneros 3, Gilberto Velázquez-Juárez 4, Fredy Geovannini Morales-Palacios 2,* and Stephanie García-Zavala 2,*

1 Department of Pharmacology, School of Exact Sciences and Engineering, University of Guadalajara, Boulevard Gral. Marcelino García Barragán 1421, Olímpica, Guadalajara 44430, Jalisco, Mexico

2 Instituto de Investigaciones Químico-Biológicas, Universidad Michoacana de San Nicolás de Hidalgo, Francisco J. Múgica, s/n, Morelia 58030, Michoacán, Mexico

3 Department of Electrophotonics, School of Exact Sciences and Engineering, University of Guadalajara, Boule-vard Gral. Marcelino García Barragán 1421, Olímpica, Guadalajara 44430, Jalisco, Mexico

4 Department of Chemistry, School of Exact Sciences and Engineering, University of Guadalajara, Boulevard Gral. Marcelino García Barragán 1421, Olímpica, Guadalajara 44430, Jalisco, Mexico

* Correspondence: geovannini.morales@umich.mx (F.G.M.-P.); 1106886k@umich.mx (S.G.-Z.)

SUPPLEMENTARY INFORMATION

TABLE OF CONTENTS

1. Analytical data of (+)-Medicarpin derivative **2**……….....……………………….……………………………..2
2. NMR, mass, and infrared spectra………………………………………………………………………...2
3. Crystal Packing and Hirshfeld Surface of conformer **1**(**II**)………………………………………………6
4. X-ray crystallographic data of conformer **1**(**II**)…………………………………………………………...8
5. Crystal Packing and Hirshfeld Surface of derivative **2**…………………………………………………....19
6. X-ray crystallographic data of derivative **2**…………………………………………………...…………22
7. DFT Optimization Coordinates for Conformational Analysis of **1**(**I**) and **1**(**II**)………………………………. 31

1. Analytical Data of (+)-Medicarpin derivative 2

a. (6aS,11aS)-9-methoxy-6a,11a-dihydro-6H-benzofuro[3,2-c]chromen-3-yl 4-nitrobenzoate (2).

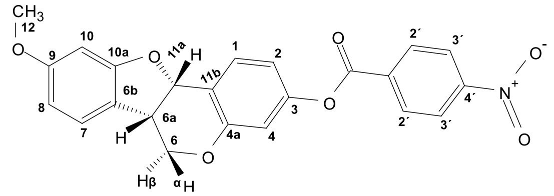

^{1}H NMR (400 MHz, CDCl$_3$): δ 3.61 (m, 1H, H-6a), 3.67 (dt, *J* = 11.1, 3.2 Hz, 1H, H-6β), 3.79 (s, 3H, OMe), 4.31 (dd, *J* = 12.6, 5.6 Hz, 1H, H-6α), 5.55 (dd, *J* = 9.8, 7.0 Hz, 1H, H-11a), 6.48 (s, 1H, H-10), 6.50 (d, *J* = 2.3 Hz, 1H, H-8), 6.86 (d, *J* = 2.0 Hz, 1H, H-4), 6.95 (dd, *J* = 8.4, 2.4 Hz, 1H, H-2), 7.62 (d, *J* = 8.7 Hz, 1H, H-1), 8.38 (s, 4H, H-1´,2´,3´,4´-BzO).

^{13}C NMR (101 MHz, CDCl$_3$): δ 39.4 (C-6a), 55.4 (OMe), 66.6 (C-6), 77.8 (C-11a), 96.9 (C-10), 106.5 (C-8), 110.6 (C-4), 114.9 (C-2), 118.4 (C-11b), 118.6 (C-6b), 123.6 (C-3´), 124.7 (C-7), 131.3 (C-2´), 132.0 (C-1), 134.6 (C-1´), 150.8 (C-4´), 151.3 (C-10a), 156.3 (C-9), 160.4 (C-4a), 161.1 (C-3), 163.0 (C=O).

IR (Ʋmax): 3110 to 3053 cm^{-1} (C-H aromatic), 2970 to 2889 cm^{-1} (low-intensity peaks, methyl C-H), 1734 cm^{-1} (acyl C=O), 1618, 1518, and 1494 cm^{-1} (medium-intensity peaks, aromatic C=C stretching), 1594 cm^{-1} (medium-intensity peak, asymmetric N-O), 1261 cm^{-1} (acyl O=C-O), 1149 cm^{-1} (medium-intensity peak, C-O-C).**MS [M$^+$]**: 419.0 m/z, calculated for 419 m/z ($C_{23}H_{17}O_7N_1$).

2. NMR, mass, and infrared spectra

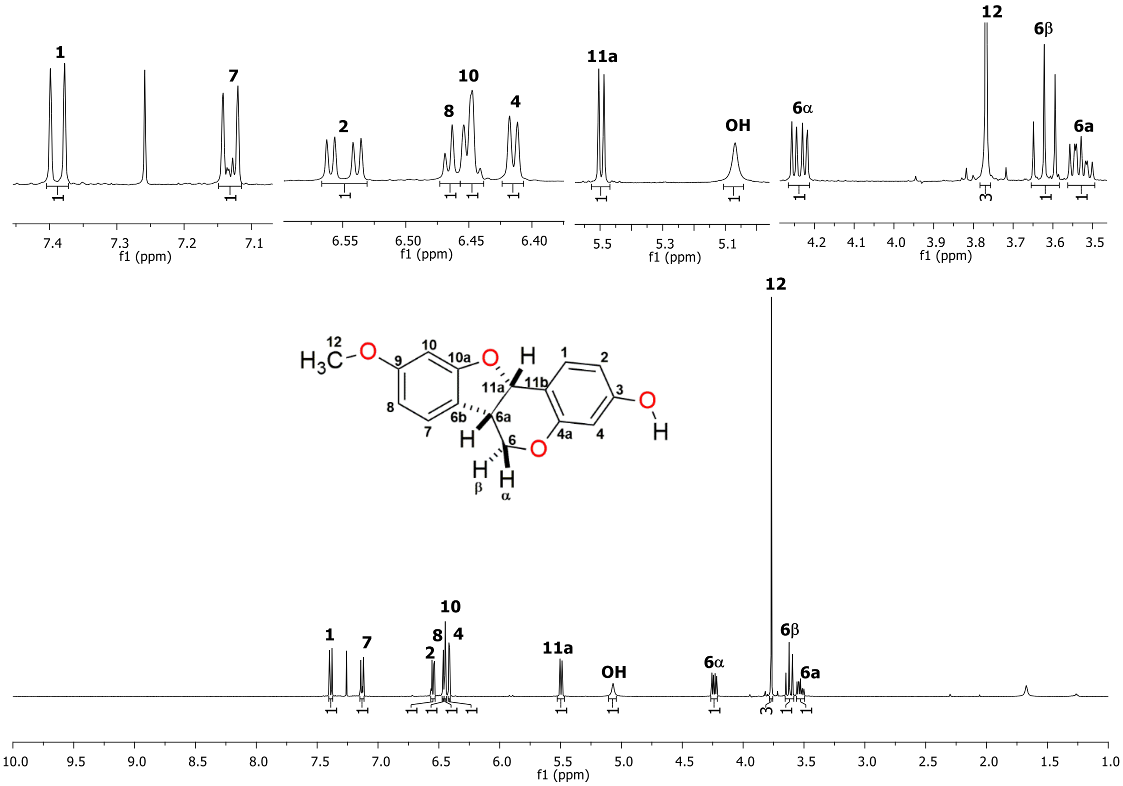

^{1}H NMR spectrum of (+)-(6a*S*,11a*S*)-medicarpin **1**(**II**) (400 MHz, CDCl$_3$).

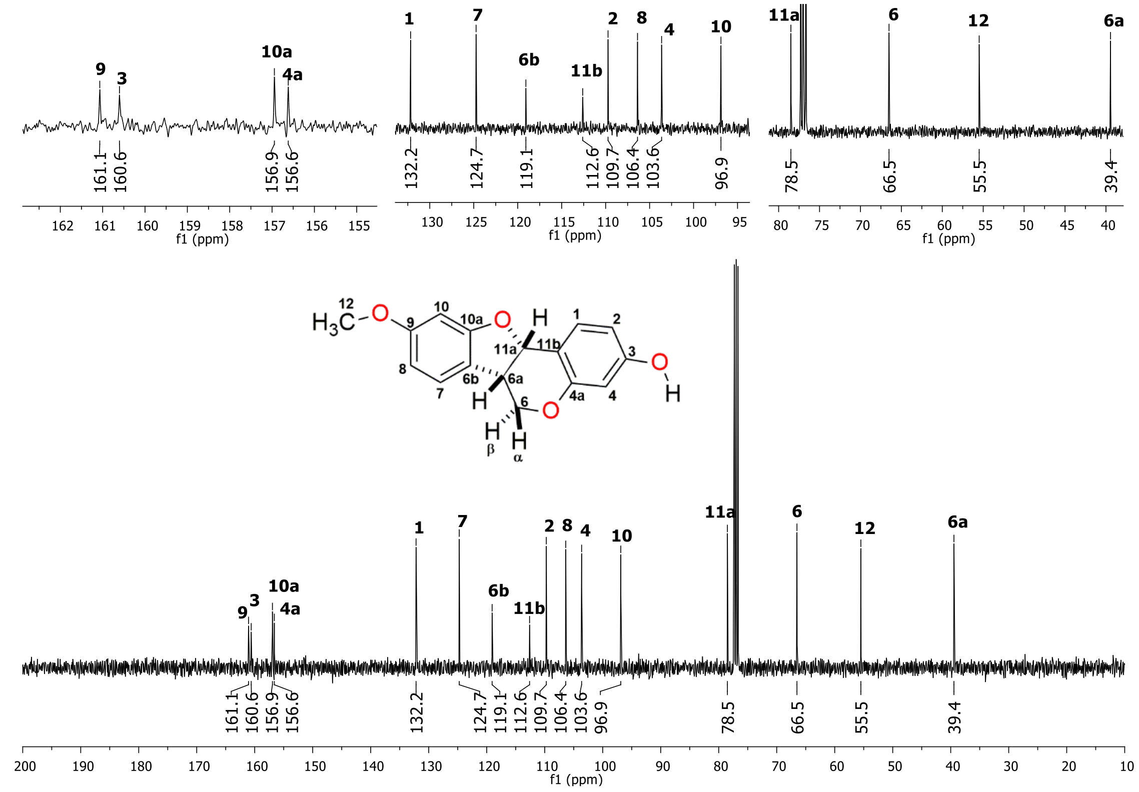

^{13}C NMR spectrum of (+)-(6a*S*,11a*S*)-medicarpin **1**(**II**) (101 MHz, $CDCl_3$).

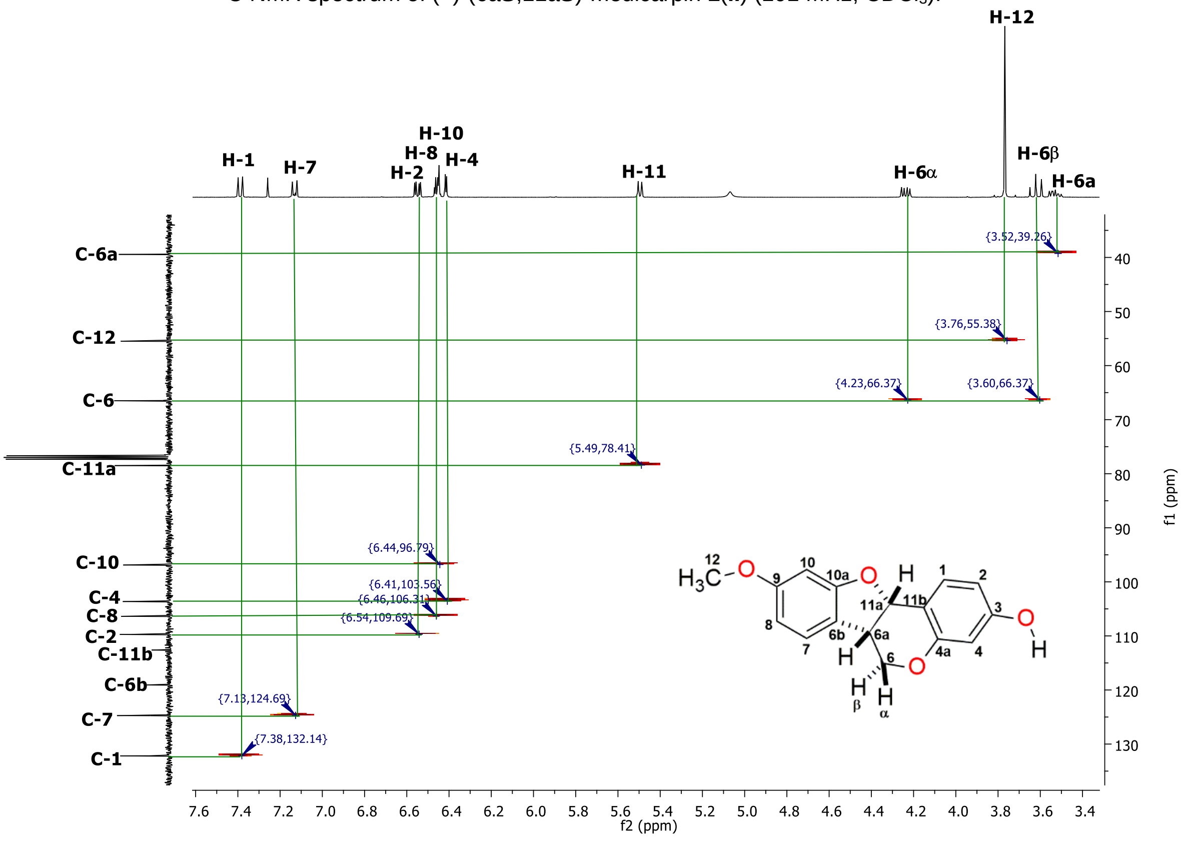

HETCOR NMR spectrum of (+)-(6a*S*, 11a*S*)-medicarpin **1**(**II**) recorded in $CDCl_3$.

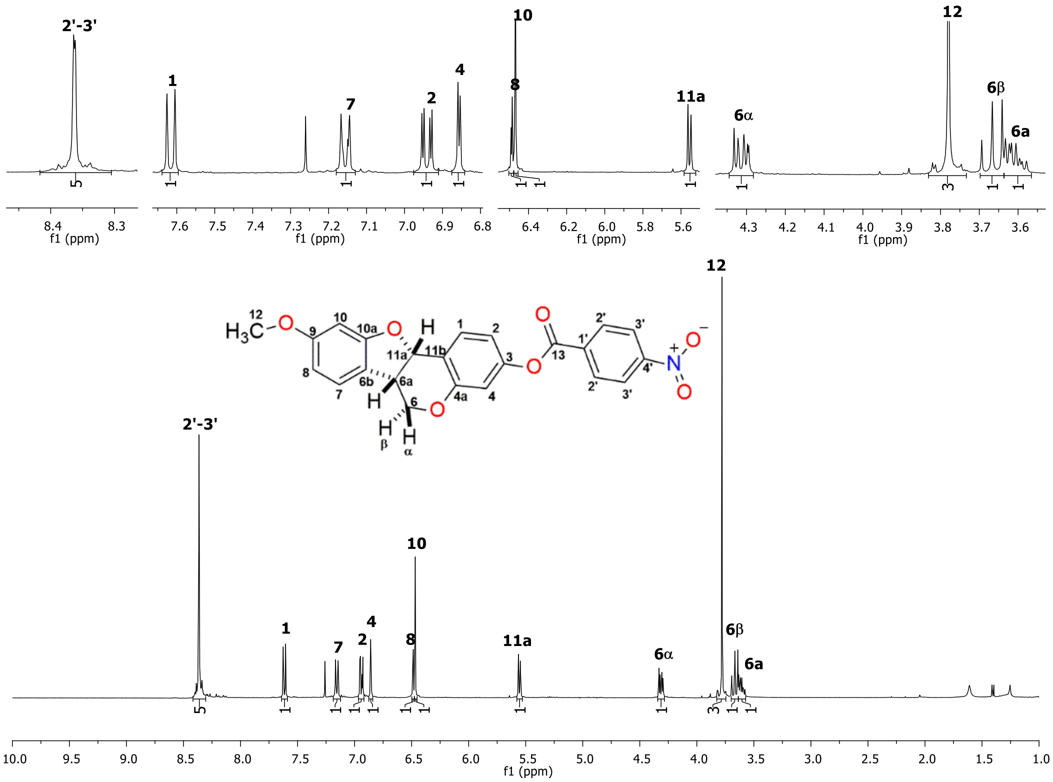

^{1}H NMR spectrum of (6a*S*,11a*S*)-9-methoxy-6a,11a-dihydro-6H-benzofuro[3,2-c]chromen-3-yl 4-nitrobenzoate (**2**) (400 MHz, $CDCl_3$).

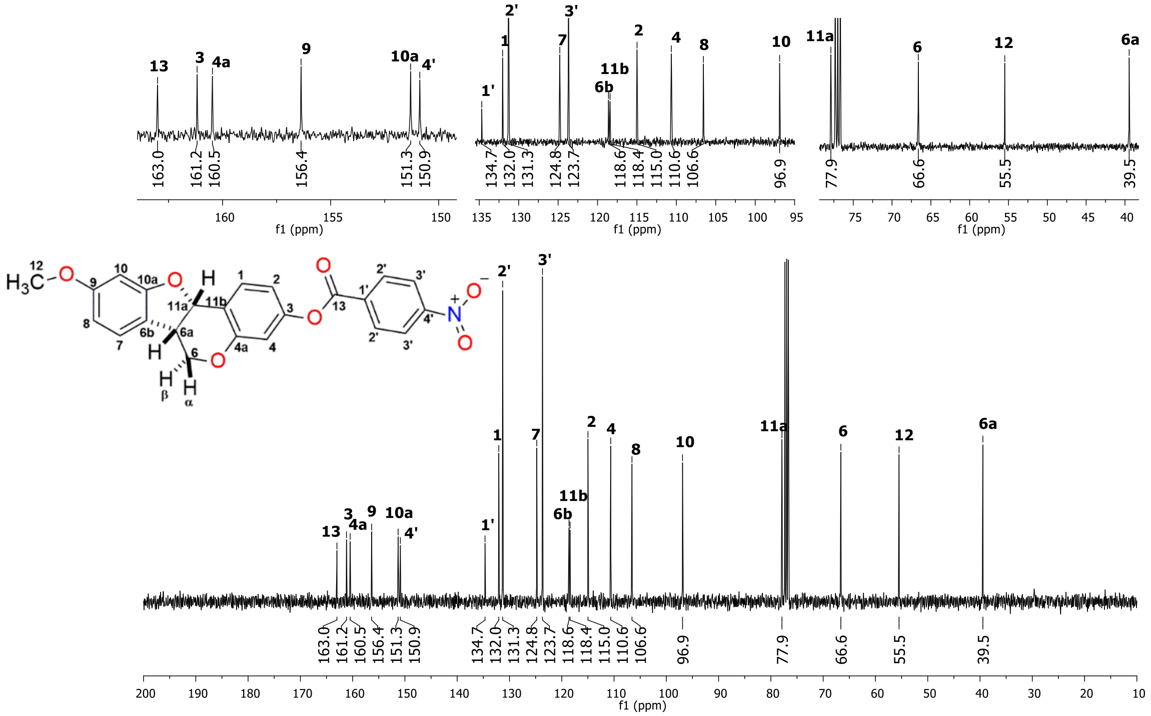

^{13}C NMR spectrum of (6a*S*,11a*S*)-9-methoxy-6a,11a-dihydro-6H-benzofuro[3,2-c]chromen-3-yl 4-nitrobenzoate (**2**) (101 MHz, $CDCl_3$).

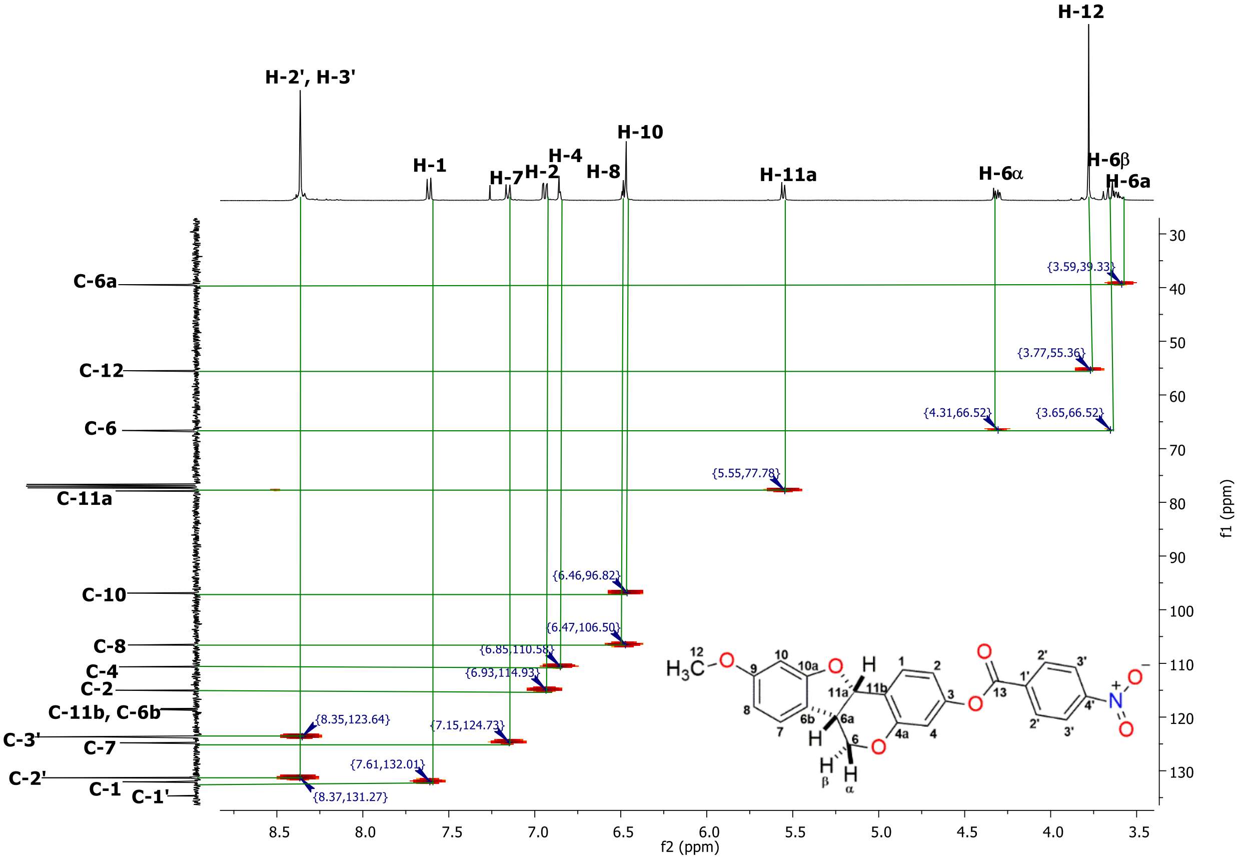

HETCOR NMR spectrum of (6a*S*,11a*S*)-9-methoxy-6a,11a-dihydro-6H-benzofuro[3,2-c]chromen-3-yl 4-nitrobenzoate (**2**) recorded in $CDCl_3$.

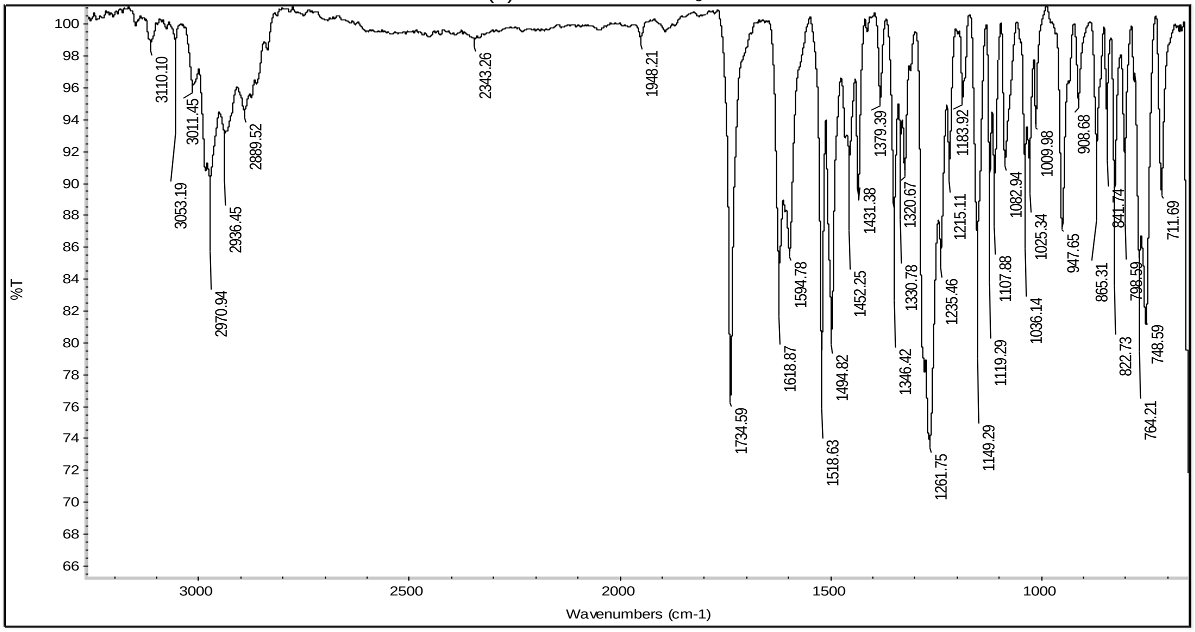

FT-IR spectrum of (6a*S*,11a*S*)-9-methoxy-6a,11a-dihydro-6H-benzofuro[3,2-c]chromen-3-yl 4-nitrobenzoate (**2**).

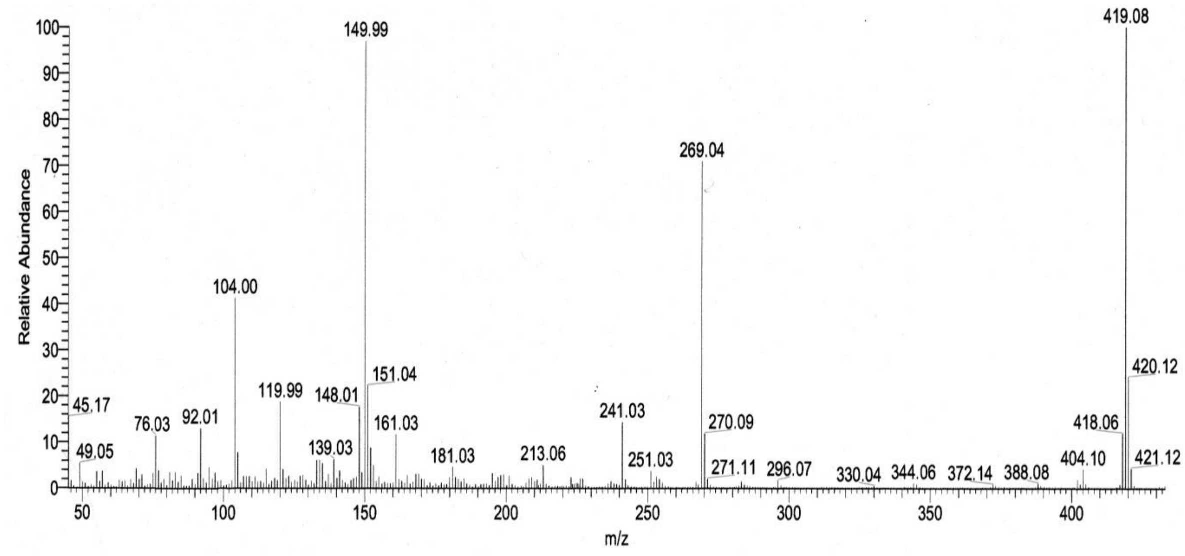

MS spectrum of (6a*S*,11a*S*)-9-methoxy-6a,11a-dihydro-6H-benzofuro[3,2-c]chromen-3-yl 4-nitrobenzoate (**2**)

3. Crystal Packing and Hirshfeld Surface of conformer 1(II).

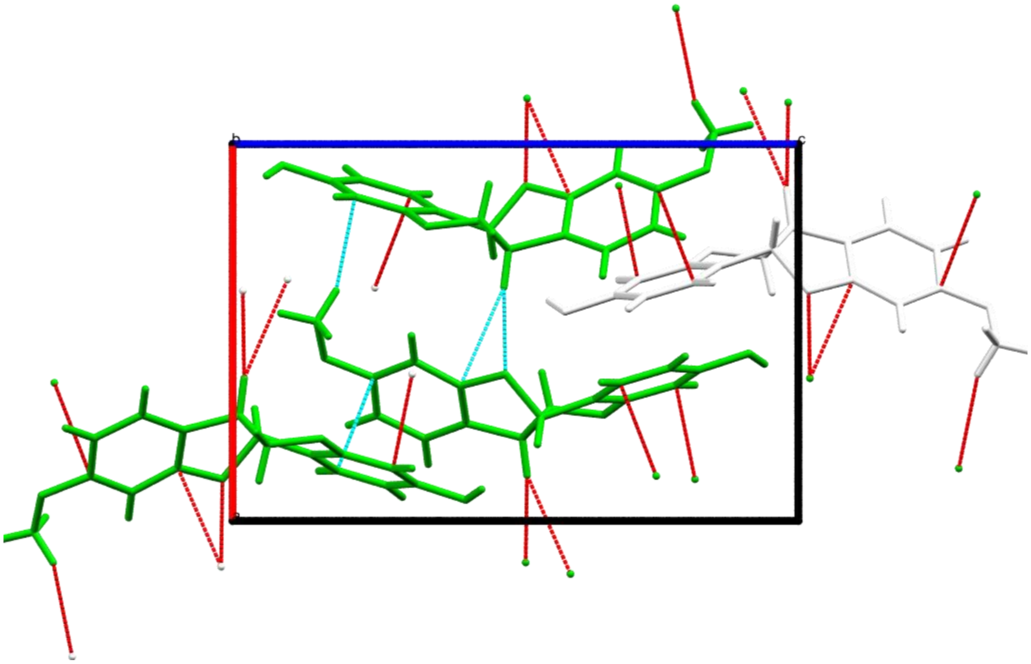

Figure S1. Full-size crystal packing image with short contact interactions shown as dashed red lines, and blue ones for H-bonding. Visualization by symmetry elements on the a-axis

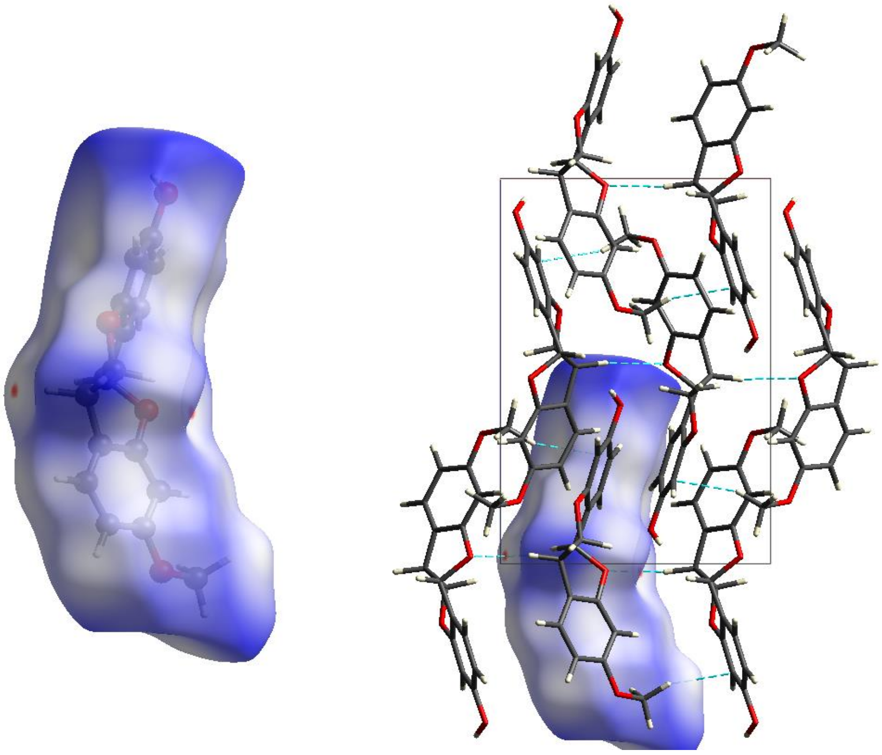

Figure S2. Hirshfeld surface image with H-bonding shown as dashed blue lines. Visualization by on the *b*-plane.

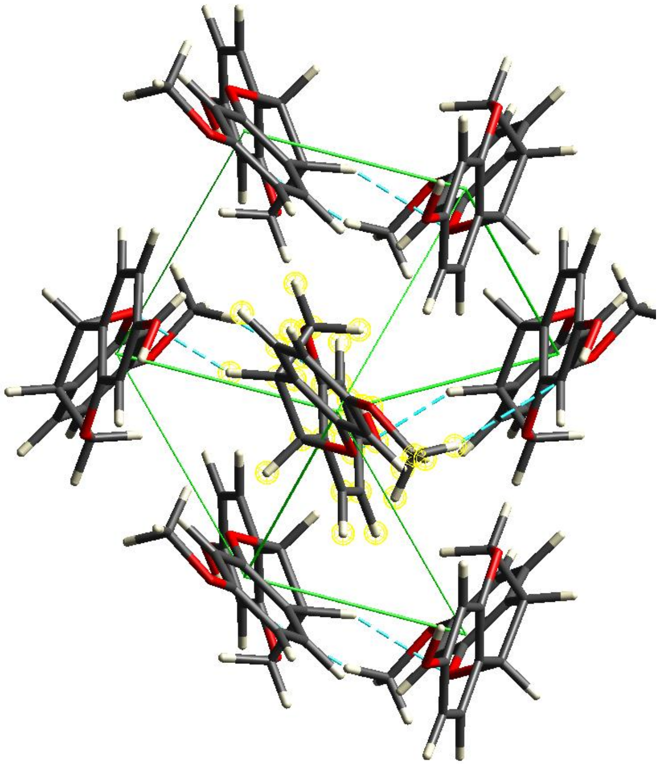

Figure S3. Energy framework dispersion diagram for a cluster of molecules of compound **1**(**II**), on the *a*-plane.

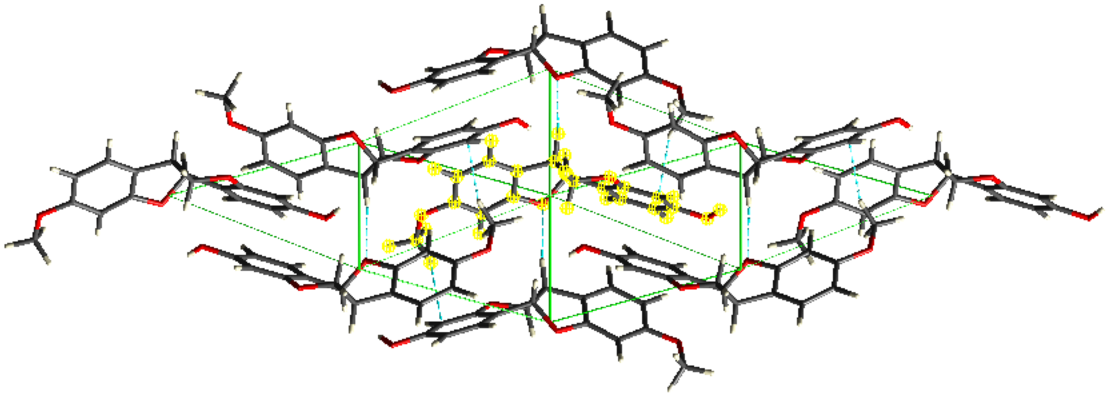

Figure S4. Energy framework dispersion diagram for a cluster of molecules of compound **1**(**II**), on the *b*-plane.

4. X-ray crystallographic data of conformer 1(II).

checkCIF/PLATON report

Structure factors have been supplied for datablock(s) shelx_xle

THIS REPORT IS FOR GUIDANCE ONLY. IF USED AS PART OF A REVIEW PROCEDURE FOR PUBLICATION, IT SHOULD NOT REPLACE THE EXPERTISE OF AN EXPERIENCED CRYSTALLOGRAPHIC REFEREE.

No syntax errors found. CIF dictionary Interpreting this report

Datablock: shelx_xle

Bond precision:	C-C = 0.0106 A		Wavelength=0.71073
Cell:	a=9.7932(4)	b=9.8680(7)	c=14.7311(4)
	alpha=90	beta=90	gamma=90
Temperature:	292 K		

	Calculated	Reported
Volume	1423.60(12)	1423.60(12)
Space group	P 21 21 21	P 21 21 21
Hall group	P 2ac 2ab	P 2ac 2ab
Moiety formula	C16 H14 O4	C16 H14 O4
Sum formula	C16 H14 O4	C16 H14 O4
Mr	270.27	270.27
Dx,g cm-3	1.261	1.261
Z	4	4

Mu (mm-1)	0.091	0.091
F000	568.0	568.0
F000’	568.32	
h,k,lmax	14,14,22	14,13,21
Nref	5119[2891]	3975
Tmin,Tmax	0.929,0.963	
Tmin’	0.913	

Correction method= Not given

Data completeness= 1.37/0.78 Theta(max)= 32.456

R(reflections)= 0.1694(3234) wR2(reflections)= 0.4568(3975)

S = 1.922 Npar= 183

The following ALERTS were generated. Each ALERT has the format **test-name_ALERT_alert-type_alert-level**. Click on the hyperlinks for more details of the test.

Alert level A

PLAT029_ALERT_3_A _diffrn_measured_fraction_theta_full value Low . 0.915 Why?

PLAT084_ALERT_3_A High wR2 Value (i.e. > 0.25) .... 0.46 Report

PLAT094_ALERT_2_A Ratio of Maximum / Minimum Residual Density .... 8.13 Report

PLAT097_ALERT_2_A Large Reported Max. (Positive) Residual Density 4.84 eA-3

PLAT971_ALERT_2_A Check Calcd Resid. Dens. 2.80Ang From O18 4.43 eA-3

Alert level B

PLAT035_ALERT_1_B _chemical_absolute_configuration Info Not Given Please Do !

PLAT082_ALERT_2_B High R1 Value .... 0.17 Report PLAT340_ALERT_3_B Low Bond Precision on C-C Bonds .... 0.01063 Ang.

PLAT420_ALERT_2_B D-H Bond Without Acceptor O18 --H18 . Please Check

PLAT934_ALERT_3_B Number of (Iobs-Icalc)/Sigma(W) > 10 Outliers .. 9 Check

4 1 0, 3 2 0, 2 4 0, 1 0 3, 2 0 5, -1 3 5,

1 3 5, 2 0 6, 0 1 9,

Alert level C

DIFMX02_ALERT_1_C The maximum difference density is > 0.1*ZMAX*0.75 The relevant atom site should be identified.

PLAT767_ALERT_4_C INS Embedded LIST 6 Instruction Should be LIST 4 Please Check

PLAT906_ALERT_3_C Large K Value in the Analysis of Variance ...... 2.506 Check

PLAT911_ALERT_3_C Missing FCF Refl Between Thmin & STh/L= 0.600 122 Report
0 2 0, 0 4 0, 0 6 0, 0 8 0, 0 10 0, 1 4 0,

---

1 5 0, 1 6 0, 1 7 0, 1 8 0, 1 9 0, 1 10 0,
1 11 0, 2 5 0, 2 6 0, 2 7 0, 2 8 0, 2 9 0,
2 10 0, 2 11 0, 3 6 0, 3 7 0, 3 8 0, 3 9 0,
3 10 0, 3 11 0, 4 7 0, 4 8 0, 4 9 0, 4 10 0,
4 11 0, 5 8 0, 5 9 0, 5 10 0, 6 9 0, 6 10 0,

7 9 0, 0 6 1, 0 7 1, 0 8 1, 0 9 1, 0 10 1,
0 11 1, 1 6 1, 1 7 1, 1 8 1, 1 9 1, 1 10 1,
1 11 1, 2 7 1, 2 8 1, 2 9 1, 2 10 1, 2 11 1,
3 7 1, 3 8 1, 3 9 1, 3 10 1, 3 11 1, 4 8 1,
4 9 1, 4 10 1, 4 11 1, 5 8 1, 5 9 1, 5 10 1,

6 9 1, 6 10 1, 0 1 2, 0 7 2, 0 8 2, 0 9 2,

0 10 2, 0 11 2, 1 8 2, 1 9 2, 1 10 2, 1 11 2,
2 8 2, 2 9 2, 2 10 2, 2 11 2, 3 8 2, 3 9 2,
3 10 2, 3 11 2, 4 8 2, 4 9 2, 4 10 2, 4 11 2,

5 9 2, 0 9 3, 0 10 3, 0 11 3, 1 9 3, 1 10 3,

PLAT915_ALERT_3_C No Flack x Check Done: Low Friedel Pair Coverage 71 %

PLAT918_ALERT_3_C Reflection(s) with I(obs) much Smaller I(calc) . 18 Check

PLAT939_ALERT_3_C Large Value of Not (SHELXL) Weight Optimized S . 21.35 Check

---

Alert level G

PLAT007_ALERT_5_G Number of Unrefined Donor-H Atoms .... 1 Report

H18

PLAT032_ALERT_4_G Std. Uncertainty on Flack Parameter Value High . 0.400 Report
PLAT063_ALERT_4_G Crystal Size Possibly too Large for Beam Size .. 1.00 mm PLAT398_ALERT_2_G Deviating C-O-C Angle From 120 for O15 . 105.7 Degree PLAT480_ALERT_4_G Long H...A H-Bond Reported H8 ..O15 . 2.65 Ang.

PLAT480_ALERT_4_G Long H...A H-Bond Reported H8 ..C14 . 2.89 Ang.

PLAT480_ALERT_4_G Long H...A H-Bond Reported H8 ..O15 . 2.65 Ang.

PLAT791_ALERT_4_G Model has Chirality at C8 (Sohncke SpGr) S Verify

PLAT791_ALERT_4_G Model has Chirality at C16 (Sohncke SpGr) R Verify PLAT883_ALERT_1_G

No Info/Value for _atom_sites_solution_primary . Please Do !

PLAT910_ALERT_3_G Missing # of FCF Reflection(s) Below Theta(Min). 4 Note

1 1 0, 0 1 1, 1 0 1, 0 0 2,

PLAT912_ALERT_4_G Missing # of FCF Reflections Above STh/L= 0.600 331 Note

PLAT916_ALERT_2_G Hooft y and Flack x Parameter Values Differ by . 0.50 Check

PLAT941_ALERT_3_G Average HKL Measurement Multiplicity .... 3.3 Low

PLAT969_ALERT_5_G The 'Henn et al.' R-Factor-gap value .... 16.293 Note

Predicted wR2: Based on SigI**2 2.80 or SHELX Weight 23.76

PLAT978_ALERT_2_G Number C-C Bonds with Positive Residual Density. 3 Info

---

5 **ALERT level A** = Most likely a serious problem - resolve or explain

5 **ALERT level B** = A potentially serious problem, consider carefully

7 **ALERT level C** = Check. Ensure it is not caused by an omission or oversight

16 **ALERT level G** = General information/check it is not something unexpected

3 ALERT type 1 CIF construction/syntax error, inconsistent or missing data

8 ALERT type 2 Indicator that the structure model may be wrong or deficient

11 ALERT type 3 Indicator that the structure quality may be low

9 ALERT type 4 Improvement, methodology, query or suggestion

2 ALERT type 5 Informative message, check

It is advisable to attempt to resolve as many as possible of the alerts in all categories. Often the minor alerts point to easily fixed oversights, errors and omissions in your CIF or refinement strategy, so attention to these fine details can be worthwhile. In order to resolve some of the more serious problems it may be necessary to carry out additional measurements or structure refinements. However, the purpose of your study may justify the reported deviations and the more serious of these should normally be commented upon in the discussion or experimental section of a paper or in the "special_details" fields of the CIF. checkCIF was carefully designed to identify outliers and unusual parameters, but every test has its limitations and alerts that are not important in a particular case may appear. Conversely, the absence of alerts does not guarantee there are no aspects of the results needing attention. It is up to the individual to critically assess their own results and, if necessary, seek expert advice.

Publication of your CIF in IUCr journals

A basic structural check has been run on your CIF. These basic checks will be run on all CIFs submitted for publication in IUCr journals (*Acta Crystallographica*, *Journal of Applied Crystallography*, *Journal of Synchrotron Radiation*); however, if you intend to submit to *Acta Crystallographica Section C* or *E* or *IUCrData*, you should make sure that full publication checks are run on the final version of your CIF prior to submission.

Publication of your CIF in other journals

Please refer to the *Notes for Authors* of the relevant journal for any special instructions relating to CIF submission.

Validation response form

Please find below a validation response form (VRF) that can be filled in and pasted into your CIF.

start Validation Reply Form

_vrf_PLAT029_shelx_xle

;

PROBLEM: _diffrn_measured_fraction_theta_full value Low . 0.915 Why?

RESPONSE: The measured data completeness fraction (0.915) for the medicarpin compound is slightly below the ideal threshold of 0.95, which is generally desirable for crystallographic studies. This limitation could be attributed to experimental factors, such as the use of the Oxford Diffraction Xcalibur S diffractometer at room temperature, where, for the orthorhombic system, only about 90% of the Ewald sphere is typically accessible. Additionally, a small number of low-angle reflections, either obscured by the beam-stop or showing poor agreement, were excluded during the final refinement cycles based on quality criteria.

;

_vrf_PLAT084_shelx_xle

;

PROBLEM: High wR2 Value (i.e. > 0.25) .... 0.46 Report

RESPONSE: The crystals exhibited very weak diffraction. Several efforts were made to grow crystals with improved diffraction quality. Data were collected at different facilities using Mo and Cu radiation sources. Although the results, especially from Mo data collection, were consistent with the model presented in this report, significant issues arose due to weak diffraction and disorder in atomic positions. The high wR2 factor is attributed to the weak diffraction and the inclusion of reflections that were essentially unobserved.

;

_vrf_PLAT094_shelx_xle

;

PROBLEM: Ratio of Maximum / Minimum Residual Density .... 8.13 Report

RESPONSE: The high ratio (8.13) is influenced by significant positive and negative residual density peaks (4.840 and -0.595 eÅ^3, respectively), arising from the weakly diffracting nature of the crystals. This structure is reported in order to support the isolation of the envelope conformation of dihydro-2H-pyran ring, based on available data.

;

_vrf_PLAT097_shelx_xle

;

PROBLEM: Large Reported Max. (Positive) Residual Density 4.84 eA-3

RESPONSE: The structure is presented to support the isolation of the envelope conformation of the dihydro-2H-pyran ring. The reported large positive residual density (4.84 eÅ^3) arises from the analysis of

difference maps based on the available data. Despite this, the results remain consistent with the overall model, and the observed residuals are being carefully considered in the context of the weak diffraction and potential disorder in atomic positions.

;

_vrf_PLAT971_shelx_xle

;

PROBLEM: Check Calcd Resid. Dens. 2.80Ang From O18 4.43 eA-3

RESPONSE: The observed residual density (4.43 eÅ³) located 2.80 Å from the O18 atom could be related to the limitations in diffraction data. Further supramolecular analysis of the electron density in this region was taken to address this discrepancy and improve the model accuracy around the oxygen atom.

;

_vrf_PLAT035_shelx_xle

;

PROBLEM: _chemical_absolute_configuration Info Not Given Please Do !

RESPONSE: The Flack parameter [x = 0.2(4)] cannot be reliably interpreted because the structure contains only light atoms (C, H, O), whose anomalous scattering is too weak to provide a clear absolute configuration signal. This makes the Flack parameter statistically indeterminate. Therefore, the absolute configuration was not derived from the diffraction data but assigned based on the known enantiopure nature of the sample. Additional experimental evidence from polarimetry and HPLC confirmed the (+)-(6aS,11aS)-medicarpin configuration.

;

_vrf_PLAT082_shelx_xle

;

PROBLEM: High R1 Value .... 0.17 Report

RESPONSE: The crystals exhibited very weak diffraction. The Mo data collection provided the most reliable data. However, issues arose in all cases due to weak diffraction elevating R1 value as a consequence of both weak diffraction and the inclusion of reflections that were essentially unobserved. Additionally, the room temperature during the data collection may have contributed to these problems, further affecting the accuracy of the refinement.

;

_vrf_PLAT340_shelx_xle

;

PROBLEM: Low Bond Precision on C-C Bonds .... 0.01063 Ang.

RESPONSE: Low resolution and relatively imprecise diffraction data due to the challenging nature of the crystal.

;

_vrf_PLAT420_shelx_xle

;

PROBLEM: D-H Bond Without Acceptor O18 --H18 . Please Check

RESPONSE: This problem is provided by the observed residual density related to the limitation in diffraction data and was resolved within further supramolecular studies.

;

_vrf_PLAT934_shelx_xle

;

PROBLEM: Number of (Iobs-Icalc)/Sigma(W) > 10 Outliers .. 9 Check

RESPONSE: Some of the reflections with high errors were omitted during integration and refinement.

;

end Validation Reply Form

;

PLATON version of 15/07/2024; check.def file version of 15/07/2024

Datablock shelx_xle - ellipsoid plot

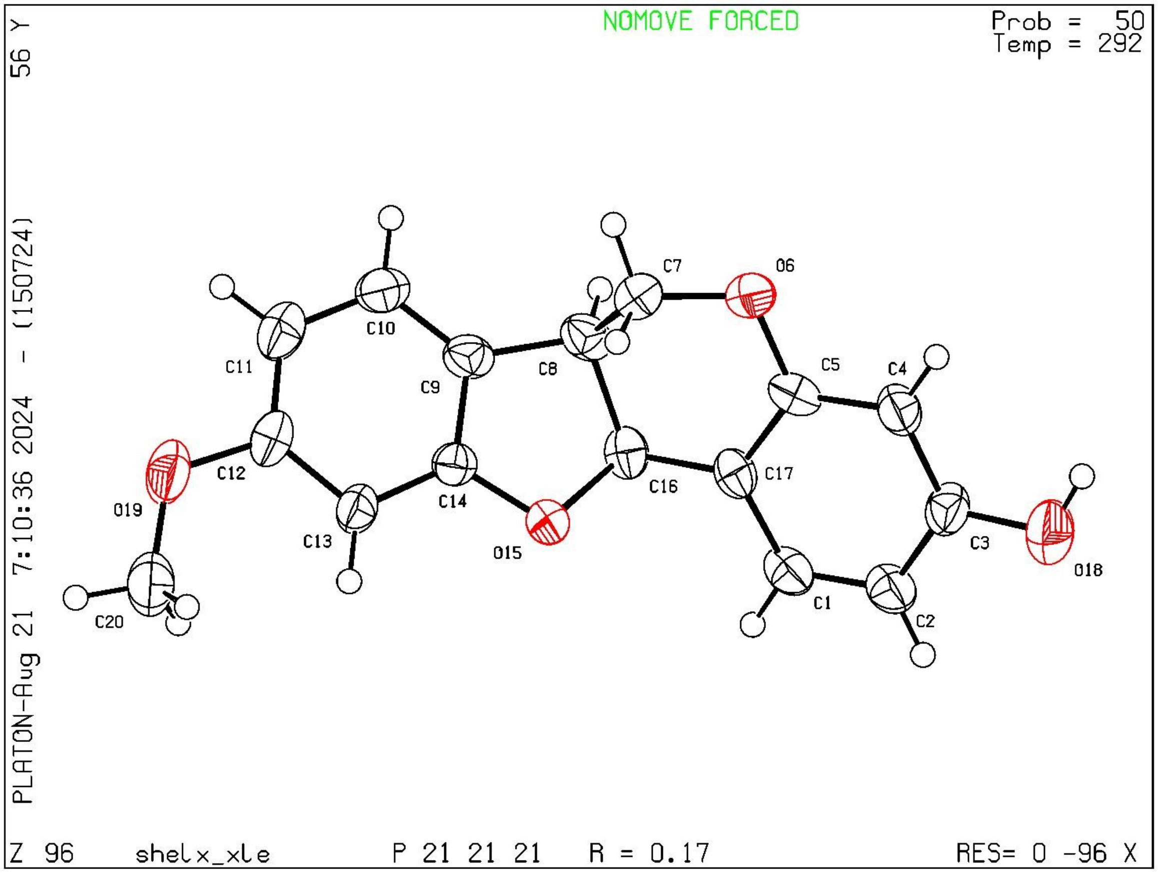

Table S1. Atomic coordinates (x 10^4) and equivalent isotropic displacement parameters ($Å^2$x 10^3) for **1**(**II**). U(eq) is defined as one third of the trace of the orthogonalized U^{ij} tensor.

	x	y	z	U(eq)
O(15)	3893(6)	923(6)	10180(3)	39(1)
O(19)	4324(8)	1934(9)	13379(4)	61(2)
O(18)	4447(10)	1172(9)	5876(4)	68(2)
C(1)	3665(8)	53(9)	8153(5)	39(2)
O(6)	2814(7)	3671(7)	8436(3)	50(2)
C(14)	3589(6)	1596(8)	10972(4)	32(1)
C(9)	2587(7)	2572(9)	10869(4)	38(2)
C(16)	2747(7)	1231(8)	9544(4)	35(1)
C(17)	3237(6)	1247(8)	8599(4)	33(1)
C(4)	3608(8)	2467(9)	7164(4)	41(2)
C(2)	4062(9)	50(9)	7267(5)	43(2)
C(13)	4236(7)	1334(9)	11784(4)	39(2)
C(12)	3809(8)	2114(9)	12513(4)	41(2)
C(7)	2970(9)	3747(10)	9384(5)	47(2)
C(5)	3219(7)	2443(8)	8069(5)	37(2)
C(11)	2803(9)	3085(10)	12445(4)	47(2)
C(8)	2205(7)	2628(9)	9879(4)	40(2)
C(10)	2186(8)	3310(9)	11602(5)	44(2)
C(3)	4010(9)	1229(9)	6764(4)	42(2)
C(20)	5290(11)	865(14)	13513(5)	61(3)

Table S2. Bond lengths [Å] and angles [°] for **1**(**II**).

O(15)-C(14)	1.374(8)	C(20)-H(20A)	0.9600
O(15)-C(16)	1.494(8)	C(20)-H(20B)	0.9600
O(19)-C(12)	1.383(8)	C(20)-H(20C)	0.9600
O(19)-C(20)	1.430(13)	C(14)-O(15)-C(16)	105.7(5)
O(18)-C(3)	1.377(9)	C(12)-O(19)-C(20)	117.6(6)
O(18)-H(18)	0.8200	C(3)-O(18)-H(18)	109.5
C(1)-C(2)	1.363(11)	C(2)-C(1)-C(17)	122.1(7)
C(1)-C(17)	1.413(12)	C(2)-C(1)-H(1)	118.9
C(1)-H(1)	0.9300	C(17)-C(1)-H(1)	118.9
O(6)-C(5)	1.384(11)	C(5)-O(6)-C(7)	113.8(6)
O(6)-C(7)	1.406(9)	O(15)-C(14)-C(13)	123.1(6)
C(14)-C(13)	1.379(8)	O(15)-C(14)-C(9)	113.5(5)
C(14)-C(9)	1.383(10)	C(13)-C(14)-C(9)	123.5(6)
C(9)-C(10)	1.360(10)	C(10)-C(9)-C(14)	119.4(6)
C(9)-C(8)	1.507(9)	C(10)-C(9)-C(8)	132.6(7)
C(16)-C(17)	1.473(8)	C(14)-C(9)-C(8)	107.9(6)
C(16)-C(8)	1.558(12)	C(17)-C(16)-O(15)	110.5(5)
C(16)-H(16)	0.9800	C(17)-C(16)-C(8)	113.6(6)
C(17)-C(5)	1.415(10)	O(15)-C(16)-C(8)	103.7(5)
C(4)-C(5)	1.387(10)	C(17)-C(16)-H(16)	109.6
C(4)-C(3)	1.412(12)	O(15)-C(16)-H(16)	109.6
C(4)-H(4)	0.9300	C(8)-C(16)-H(16)	109.6
C(2)-C(3)	1.380(11)	C(1)-C(17)-C(5)	116.3(6)
C(2)-H(2)	0.9300	C(1)-C(17)-C(16)	121.8(6)
C(13)-C(12)	1.386(9)	C(5)-C(17)-C(16)	121.8(7)
C(13)-H(13)	0.9300	C(5)-C(4)-C(3)	117.5(7)
C(12)-C(11)	1.379(13)	C(5)-C(4)-H(4)	121.2
C(7)-C(8)	1.521(12)	C(3)-C(4)-H(4)	121.2
C(7)-H(7A)	0.9700	C(1)-C(2)-C(3)	120.1(7)
C(7)-H(7B)	0.9700	C(1)-C(2)-H(2)	119.9
C(11)-C(10)	1.399(11)	C(3)-C(2)-H(2)	119.9
C(11)-H(11)	0.9300	C(14)-C(13)-C(12)	115.4(7)
C(8)-H(8)	0.9800	C(14)-C(13)-H(13)	122.3
C(10)-H(10)	0.9300	C(12)-C(13)-H(13)	122.3

C(11)-C(12)-O(19)	114.6(6)	C(7)-C(8)-C(16)	108.8(6)
C(11)-C(12)-C(13)	123.1(7)	C(9)-C(8)-H(8)	111.6
O(19)-C(12)-C(13)	122.2(8)	C(7)-C(8)-H(8)	111.6
O(6)-C(7)-C(8)	112.6(7)	C(16)-C(8)-H(8)	111.6
O(6)-C(7)-H(7A)	109.1	C(9)-C(10)-C(11)	119.7(8)
C(8)-C(7)-H(7A)	109.1	C(9)-C(10)-H(10)	120.2
O(6)-C(7)-H(7B)	109.1	C(11)-C(10)-H(10)	120.2
C(8)-C(7)-H(7B)	109.1	O(18)-C(3)-C(2)	117.6(7)
H(7A)-C(7)-H(7B)	107.8	O(18)-C(3)-C(4)	121.2(7)
O(6)-C(5)-C(4)	116.1(7)	C(2)-C(3)-C(4)	121.1(6)
O(6)-C(5)-C(17)	121.2(6)	O(19)-C(20)-H(20A)	109.5
C(4)-C(5)-C(17)	122.8(7)	O(19)-C(20)-H(20B)	109.5
C(12)-C(11)-C(10)	118.9(7)	H(20A)-C(20)-H(20B)	109.5
C(12)-C(11)-H(11)	120.5	O(19)-C(20)-H(20C)	109.5
C(10)-C(11)-H(11)	120.5	H(20A)-C(20)-H(20C)	109.5
C(9)-C(8)-C(7)	111.6(7)	H(20B)-C(20)-H(20C)	109.5
C(9)-C(8)-C(16)	100.9(6)		

Table S3 Hydrogen bonds for **1**(**II**) [Å and °].

D-H...A	d(D-H)	d(H...A)	d(D...A)	<(DHA)
C(8)-H(8)...O(15)#1	0.98	2.65	3.546(9)	152.8
C(8)-H(8)...C(14)#1	0.98	2.89	3.833(9)	161.3
C(8)-H(8)...O(15)#1	0.98	2.65	3.546(9)	152.8

Symmetry transformations used to generate equivalent atoms:

#1 x-1/2,-y+1/2,-z+2

Table S4 Anisotropic displacement parameters ($Å^2$x 10^3) for **1**(**II**). The anisotropic displacement factor exponent takes the form: $-2\ ^{2}[\ h^2 a^{*2} U^{11} + ... + 2\ h\ k\ a^*\ b^*\ U^{12}\ ]$

	U^{11}	U^{22}	U^{33}	U^{23}	U^{13}	U^{12}
O(15)	43(3)	43(3)	31(2)	1(2)	-2(2)	11(2)
O(19)	70(4)	82(6)	33(2)	-17(3)	-7(2)	11(4)
O(18)	101(6)	68(5)	36(2)	2(3)	8(3)	10(5)
C(1)	45(3)	28(4)	42(3)	3(2)	-14(3)	-6(3)
O(6)	64(4)	45(4)	40(2)	4(2)	9(2)	20(3)
C(14)	31(3)	31(4)	33(2)	2(2)	3(2)	1(2)
C(9)	33(3)	39(5)	42(3)	6(3)	6(2)	6(3)
C(16)	35(3)	39(4)	32(2)	1(2)	-3(2)	-8(3)
C(17)	31(2)	38(4)	31(2)	2(2)	-8(2)	-5(3)
C(4)	51(4)	39(5)	32(3)	5(3)	-5(2)	-1(3)
C(2)	60(4)	26(4)	43(3)	0(3)	-14(3)	-4(3)
C(13)	39(3)	50(5)	28(2)	-5(3)	3(2)	7(3)
C(12)	45(3)	46(5)	31(3)	-10(3)	3(2)	-6(3)
C(7)	59(4)	42(5)	38(3)	-3(3)	4(3)	13(4)
C(5)	33(3)	31(4)	46(3)	9(3)	-4(2)	2(3)
C(11)	55(4)	49(5)	37(3)	-6(3)	11(3)	-6(4)
C(8)	30(3)	47(5)	43(3)	8(3)	0(2)	6(3)
C(10)	46(4)	36(5)	48(3)	1(3)	9(3)	7(3)
C(3)	58(4)	35(5)	32(3)	-5(3)	1(3)	-5(3)
C(20)	59(5)	88(8)	36(3)	-4(4)	-1(3)	13(5)

5. Crystal Packing and Hirshfeld Surface of derivative 2.

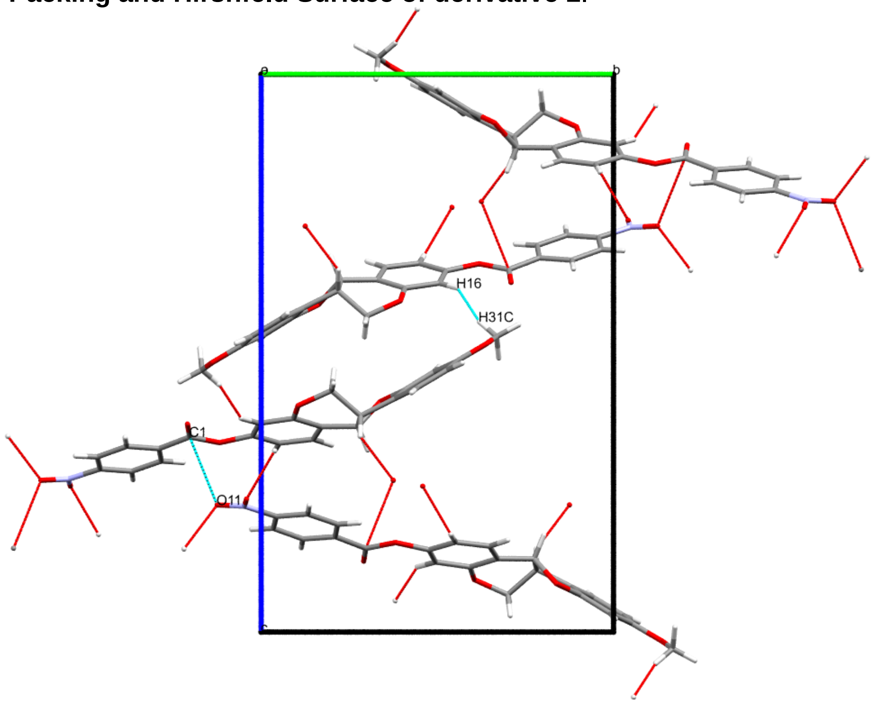

Figure S5. Full-size crystal packing image with short contact interactions shown as dashed red lines, and blue ones for H-bonding. Visualization on the *b*-axis.

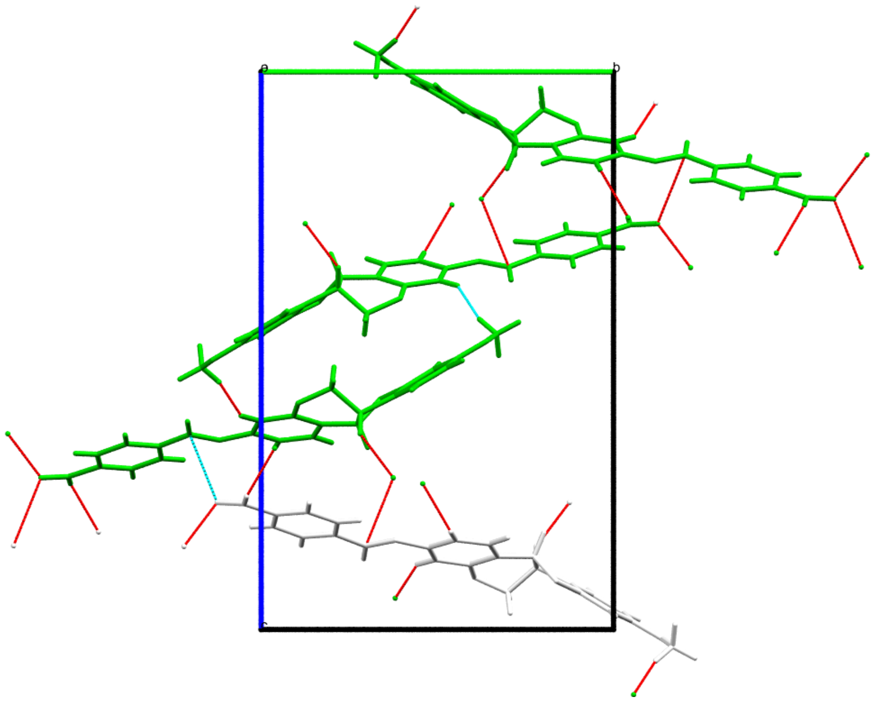

Figure S6. Full-size crystal packing image with short contact interactions shown as dashed red lines, and blue ones for H-bonding. Visualization by symmetry elements on the *b*-axis.

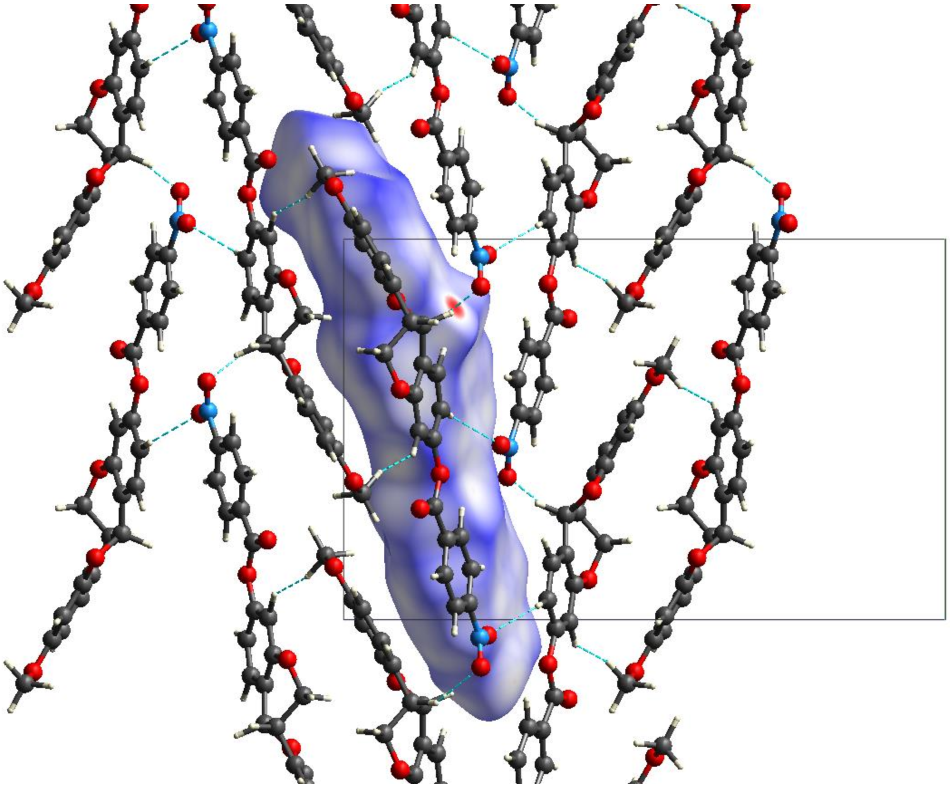

Figure S7. Hirshfeld surface image with H-bonding shown as dashed blue lines. Visualization by on the *a*-plane.

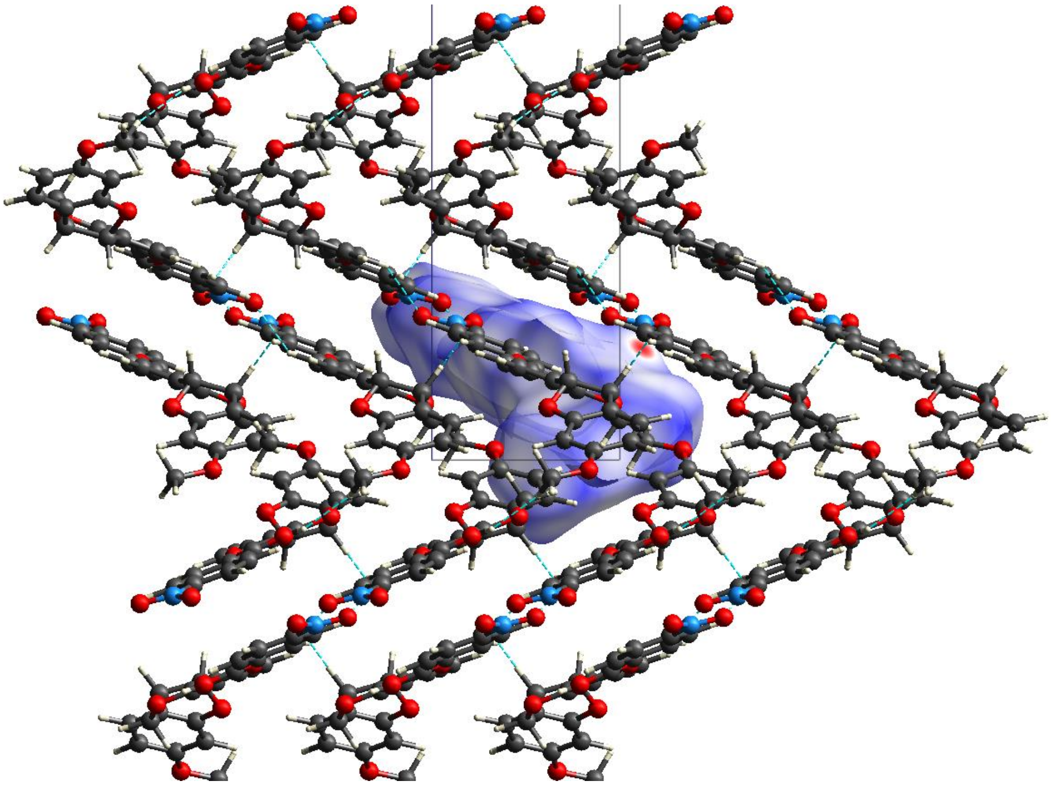

Figure S8. Hirshfeld surface image with H-bonding shown as dashed blue lines. Visualization by on the *b*-plane.

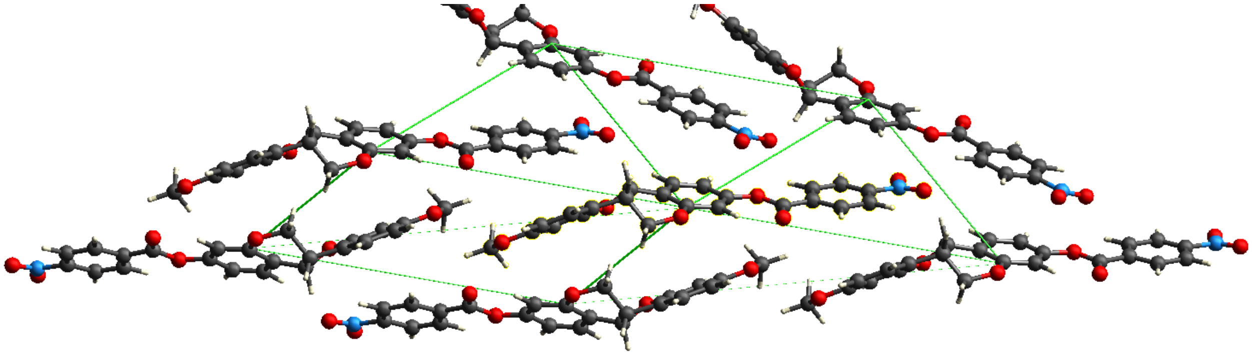

Figure S9. Energy framework dispersion diagram for a cluster of molecules of compound **2**, on the *a*-plane.

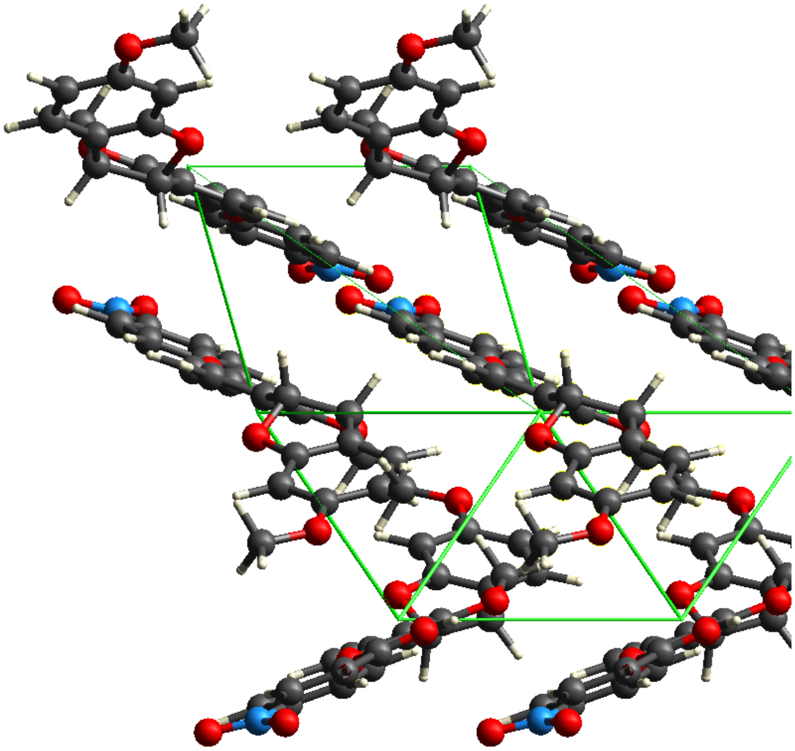

Figure S10. Energy framework dispersion diagram for a cluster of molecules of compound **2**, on the *b*-plane.

6. **X-ray crystallographic data of derivative 2**

checkCIF/PLATON report

Structure factors have been supplied for datablock(s) Medicarpin_Bz-p-NO2

THIS REPORT IS FOR GUIDANCE ONLY. IF USED AS PART OF A REVIEW PROCEDURE FOR PUBLICATION, IT SHOULD NOT REPLACE THE EXPERTISE OF AN EXPERIENCED CRYSTALLOGRAPHIC REFEREE.

No syntax errors found. CIF dictionary Interpreting this report

Datablock: Medicarpin_Bz-p-NO2

Bond precision:	C-C = 0.0041 A		Wavelength=0.71073
Cell:	a=6.6717(6)	b=13.5236(12)	c=21.3751(17)
	alpha=90	beta=90	gamma=90
Temperature:	293 K		

	Calculated	Reported
Volume	1928.6(3)	1928.6(3)
Space group	P 21 21 21	P 21 21 21
Hall group	P 2ac 2ab	P 2ac 2ab
Moiety formula	C23 H17 N O7	C23 H17 N O7
Sum formula	C23 H17 N O7	C23 H17 N O7
Mr	419.38	419.38
Dx,g cm-3	1.444	1.444
Z	4	4
Mu (mm-1)	0.108	0.108
F000	872.0	872.0
F000’	872.51	
h,k,lmax	10,20,32	9,20,30
Nref	7028[3971]	6160
Tmin,Tmax	0.949,0.963	
Tmin’	0.949	

Correction method= Not given

Data completeness= 1.55/0.88 Theta(max)= 32.593

R(reflections)= 0.0606(3770) wR2(reflections)= 0.1211(6160)

S = 1.086 Npar= 281

The following ALERTS were generated. Each ALERT has the format **test-name_ALERT_alert-type_alert-level**. Click on the hyperlinks for more details of the test.

Alert level B

PLAT035_ALERT_1_B _chemical_absolute_configuration Info Not Given Please Do !

Alert level C

PLAT340_ALERT_3_C Low Bond Precision on C-C Bonds .... 0.00413 Ang.

PLAT906_ALERT_3_C Large K Value in the Analysis of Variance ...... 6.127 Check

PLAT906_ALERT_3_C Large K Value in the Analysis of Variance ...... 2.529 Check

---

Alert level G

PLAT199_ALERT_1_G Reported _cell_measurement_temperature ..... (K) 293 Check

PLAT200_ALERT_1_G Reported _diffrn_ambient_temperature ..... (K) 293 Check

PLAT398_ALERT_2_G Deviating C-O-C Angle From 120 for O27 . 105.6 Degree

PLAT791_ALERT_4_G Model has Chirality at C20 (Sohncke SpGr) S Verify

PLAT791_ALERT_4_G Model has Chirality at C28 (Sohncke SpGr) S Verify PLAT883_ALERT_1_G Absent Datum for _atom_sites_solution_primary .. Please Do !

PLAT910_ALERT_3_G Missing # of FCF Reflection(s) Below Theta(Min). 4 Note

0 2 0, 0 1 1, 0 0 2, 0 1 2,

PLAT912_ALERT_4_G Missing # of FCF Reflections Above STh/L= 0.600 307 Note

PLAT933_ALERT_2_G Number of HKL-OMIT Records in Embedded .res File 2 Note

0 12 1, -5 4 21,

PLAT941_ALERT_3_G Average HKL Measurement Multiplicity .... 3.2 Low

PLAT952_ALERT_5_G Calculated (ThMax) and CIF-Reported Lmax Differ. 2 Units

PLAT958_ALERT_1_G Calculated (ThMax) and Actual (FCF) Lmax Differ. 2 Units

PLAT969_ALERT_5_G The 'Henn et al.' R-Factor-gap value .... 2.537 Note

Predicted wR2: Based on SigI**2 4.78 or SHELX Weight 11.16

PLAT978_ALERT_2_G Number C-C Bonds with Positive Residual Density. 0 Info PLAT992_ALERT_5_G Repd & Actual _reflns_number_gt Values Differ by 2 Check

---

0 **ALERT level A** = Most likely a serious problem - resolve or explain
1 **ALERT level B** = A potentially serious problem, consider carefully
3 **ALERT level C** = Check. Ensure it is not caused by an omission or oversight
15 **ALERT level G** = General information/check it is not something unexpected

5 ALERT type 1 CIF construction/syntax error, inconsistent or missing data
3 ALERT type 2 Indicator that the structure model may be wrong or deficient
5 ALERT type 3 Indicator that the structure quality may be low 3 ALERT type 4 Improvement, methodology, query or suggestion
3 ALERT type 5 Informative message, check

Validation response form

Please find below a validation response form (VRF) that can be filled in and pasted into your CIF.

start Validation Reply Form
_vrf_PLAT035_Medicarpin_Bz-p-NO2
; PROBLEM: _chemical_absolute_configuration Info Not Given Please Do !
RESPONSE: ...
;
_vrf_PLAT340_Medicarpin_Bz-p-NO2
; PROBLEM: Low Bond Precision on C-C Bonds .... 0.00413 Ang.
RESPONSE: ...
;
_vrf_PLAT906_Medicarpin_Bz-p-NO2
;
PROBLEM: Large K Value in the Analysis of Variance ...... 6.127 Check RESPONSE: ...
;
end Validation Reply Form

It is advisable to attempt to resolve as many as possible of the alerts in all categories. Often the minor alerts point to easily fixed oversights, errors and omissions in your CIF or refinement strategy, so attention

to these fine details can be worthwhile. In order to resolve some of the more serious problems it may be necessary to carry out additional measurements or structure refinements. However, the purpose of your study may justify the reported deviations and the more serious of these should normally be commented upon in the discussion or experimental section of a paper or in the "special_details" fields of the CIF. checkCIF was carefully designed to identify outliers and unusual parameters, but every test has its limitations and alerts that are not important in a particular case may appear. Conversely, the absence of alerts does not guarantee there are no aspects of the results needing attention. It is up to the individual to critically assess their own results and, if necessary, seek expert advice.

Publication of your CIF in IUCr journals

A basic structural check has been run on your CIF. These basic checks will be run on all CIFs submitted for publication in IUCr journals (*Acta Crystallographica*, *Journal of Applied Crystallography*, *Journal of Synchrotron Radiation*); however, if you intend to submit to *Acta Crystallographica Section C* or *E* or *IUCrData*, you should make sure that full publication checks are run on the final version of your CIF prior to submission.

Publication of your CIF in other journals

Please refer to the *Notes for Authors* of the relevant journal for any special instructions relating to CIF submission.

PLATON version of 11/11/2024; check.def file version of 11/11/2024

Datablock Medicarpin_Bz-p-NO2 - ellipsoid plot

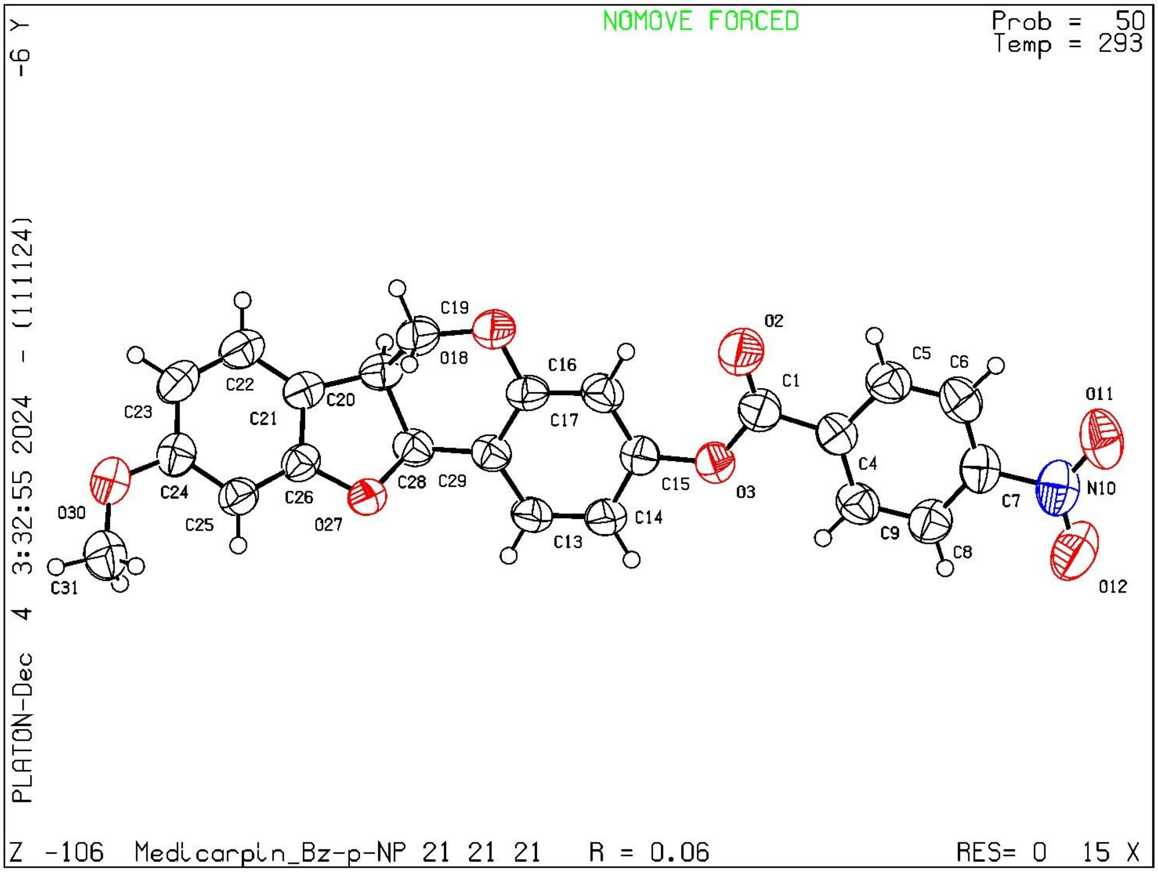

Table S5. Atomic coordinates (x 10^4) and equivalent isotropic displacement parameters (Å^{2}x 10^3) for **2**. U(eq) is defined as one third of the trace of the orthogonalized U^{ij} tensor.

	x	y	z	U(eq)
O(3)	4498(3)	3819(1)	8396(1)	62(1)
O(27)	6176(3)	8378(1)	9131(1)	58(1)
O(30)	8339(4)	11384(1)	10166(1)	75(1)
O(18)	9625(3)	6050(1)	9061(1)	77(1)
C(29)	6481(4)	6708(2)	8683(1)	51(1)
C(13)	4590(5)	6481(2)	8459(1)	60(1)
C(26)	7557(4)	9065(2)	9338(1)	52(1)
O(2)	7117(4)	2908(2)	8730(2)	104(1)
C(4)	4300(5)	2101(2)	8289(1)	58(1)
C(25)	7050(4)	9906(2)	9666(1)	55(1)
O(12)	-518(5)	-415(2)	7615(1)	107(1)
C(20)	9474(4)	7822(2)	8877(1)	56(1)

C(21)	9508(4)	8813(2)	9192(1)	53(1)
O(11)	2200(5)	-1250(2)	7721(1)	95(1)
C(24)	8611(5)	10520(2)	9845(1)	59(1)
C(22)	11046(5)	9425(2)	9380(2)	66(1)
C(23)	10582(5)	10281(2)	9701(2)	68(1)
C(17)	7735(4)	5925(2)	8832(1)	55(1)
C(16)	7151(5)	4947(2)	8744(1)	61(1)
C(28)	7265(4)	7743(2)	8688(1)	54(1)
C(19)	9973(5)	6998(2)	9330(2)	68(1)
C(14)	3946(5)	5517(2)	8387(1)	65(1)
N(10)	1247(6)	-476(2)	7742(1)	77(1)
C(1)	5495(5)	2971(2)	8499(2)	66(1)
C(7)	2328(5)	431(2)	7926(1)	62(1)
C(9)	2359(5)	2168(2)	8067(1)	64(1)
C(6)	4244(5)	346(2)	8141(2)	70(1)
C(8)	1347(5)	1325(2)	7883(1)	68(1)
C(15)	5258(4)	4760(2)	8525(1)	53(1)
C(5)	5238(5)	1184(2)	8330(1)	68(1)
C(31)	6359(6)	11681(2)	10299(2)	89(1)

Table S6 . Bond lengths [Å] and angles [°] for **2**.

O(3)-C(1)	1.344(3)	C(4)-C(1)	1.490(4)	C(28)-H(28)	0.9800
O(3)-C(15)	1.397(3)	C(25)-C(24)	1.386(4)	C(19)-H(19A)	0.9700
O(27)-C(26)	1.381(3)	C(25)-H(25)	0.9300	C(19)-H(19B)	0.9700
O(27)-C(28)	1.470(3)	O(12)-N(10)	1.211(4)	C(14)-C(15)	1.378(4)
O(30)-C(24)	1.367(3)	C(20)-C(21)	1.500(4)	C(14)-H(14)	0.9300
O(30)-C(31)	1.410(4)	C(20)-C(19)	1.515(4)	N(10)-C(7)	1.476(4)
O(18)-C(17)	1.363(3)	C(20)-C(28)	1.532(4)	C(7)-C(6)	1.362(4)
O(18)-C(19)	1.424(3)	C(20)-H(20)	0.9800	C(7)-C(8)	1.379(4)
C(29)-C(13)	1.384(4)	C(21)-C(22)	1.378(4)	C(9)-C(8)	1.382(4)
C(29)-C(17)	1.387(4)	O(11)-N(10)	1.225(4)	C(9)-H(9)	0.9300
C(29)-C(28)	1.494(4)	C(24)-C(23)	1.389(4)	C(6)-C(5)	1.374(4)
C(13)-C(14)	1.382(4)	C(22)-C(23)	1.382(4)	C(6)-H(6)	0.9300
C(13)-H(13)	0.9300	C(22)-H(22)	0.9300	C(8)-H(8)	0.9300
C(26)-C(25)	1.379(4)	C(23)-H(23)	0.9300	C(5)-H(5)	0.9300

C(26)-C(21)	1.381(4)	C(17)-C(16)	1.392(4)	C(31)-H(31A)	0.9600
O(2)-C(1)	1.193(4)	C(16)-C(15)	1.371(4)	C(31)-H(31B)	0.9600
C(4)-C(9)	1.382(4)	C(16)-H(16)	0.9300	C(31)-H(31C)	0.9600
C(4)-C(5)	1.392(4)				

C(1)-O(3)-C(15)	124.4(2)	O(30)-C(24)-C(23)	115.8(3)	O(12)-N(10)-O(11)	123.7(3)
C(26)-O(27)-C(28)	105.6(2)	C(25)-C(24)-C(23)	120.7(3)	O(12)-N(10)-C(7)	118.6(3)
C(24)-O(30)-C(31)	118.0(3)	C(21)-C(22)-C(23)	118.7(3)	O(11)-N(10)-C(7)	117.7(3)
C(17)-O(18)-C(19)	114.0(2)	C(21)-C(22)-H(22)	120.6	O(2)-C(1)-O(3)	125.2(3)
C(13)-C(29)-C(17)	117.4(3)	C(23)-C(22)-H(22)	120.6	O(2)-C(1)-C(4)	123.6(3)
C(13)-C(29)-C(28)	122.0(2)	C(22)-C(23)-C(24)	121.2(3)	O(3)-C(1)-C(4)	111.1(3)
C(17)-C(29)-C(28)	120.2(3)	C(22)-C(23)-H(23)	119.4	C(6)-C(7)-C(8)	122.8(3)
C(14)-C(13)-C(29)	122.1(3)	C(24)-C(23)-H(23)	119.4	C(6)-C(7)-N(10)	118.6(3)
C(14)-C(13)-H(13)	119.0	O(18)-C(17)-C(29)	123.1(3)	C(8)-C(7)-N(10)	118.6(3)
C(29)-C(13)-H(13)	119.0	O(18)-C(17)-C(16)	115.2(2)	C(8)-C(9)-C(4)	120.0(3)
C(25)-C(26)-C(21)	123.4(3)	C(29)-C(17)-C(16)	121.7(3)	C(8)-C(9)-H(9)	120.0
C(25)-C(26)-O(27)	123.6(3)	C(15)-C(16)-C(17)	118.7(3)	C(4)-C(9)-H(9)	120.0
C(21)-C(26)-O(27)	113.0(2)	C(15)-C(16)-H(16)	120.7	C(7)-C(6)-C(5)	118.9(3)
C(9)-C(4)-C(5)	120.1(3)	C(17)-C(16)-H(16)	120.7	C(7)-C(6)-H(6)	120.5
C(9)-C(4)-C(1)	123.5(3)	O(27)-C(28)-C(29)	112.2(2)	C(5)-C(6)-H(6)	120.5
C(5)-C(4)-C(1)	116.4(3)	O(27)-C(28)-C(20)	105.4(2)	C(7)-C(8)-C(9)	118.2(3)
C(26)-C(25)-C(24)	116.8(3)	C(29)-C(28)-C(20)	113.8(2)	C(7)-C(8)-H(8)	120.9
C(26)-C(25)-H(25)	121.6	O(27)-C(28)-H(28)	108.4	C(9)-C(8)-H(8)	120.9
C(24)-C(25)-H(25)	121.6	C(29)-C(28)-H(28)	108.4	C(16)-C(15)-C(14)	121.4(3)
C(21)-C(20)-C(19)	111.5(2)	C(20)-C(28)-H(28)	108.4	C(16)-C(15)-O(3)	124.7(2)
C(21)-C(20)-C(28)	101.3(2)	O(18)-C(19)-C(20)	111.6(2)	C(14)-C(15)-O(3)	113.8(3)
C(19)-C(20)-C(28)	109.2(2)	O(18)-C(19)-H(19A)	109.3	C(6)-C(5)-C(4)	119.9(3)
C(21)-C(20)-H(20)	111.5	C(20)-C(19)-H(19A)	109.3	C(6)-C(5)-H(5)	120.0
C(19)-C(20)-H(20)	111.5	O(18)-C(19)-H(19B)	109.3	C(4)-C(5)-H(5)	120.0
C(28)-C(20)-H(20)	111.5	C(20)-C(19)-H(19B)	109.3	O(30)-C(31)-H(31A)	109.5
C(22)-C(21)-C(26)	19.2(3)	H(19A)-C(19)-H(19B)	108.0	O(30)-C(31)-H(31B)	109.5
C(22)-C(21)-C(20)	132.7(3)	C(15)-C(14)-C(13)	118.7(3)	H(31A)-C(31)-H(31B)	109.5
C(26)-C(21)-C(20)	107.9(2)	C(15)-C(14)-H(14)	120.7	O(30)-C(31)-H(31C)	109.5
O(30)-C(24)-C(25)	123.4(3)	C(13)-C(14)-H(14)	120.7	H(31A)-C(31)-H(31C)	109.5
				H(31B)-C(31)-H(31C)	109.5

Table S7 . Hydrogen bonds for **2** [Å and °].

D-H...A	d(D-H)	d(H...A)	d(D...A)	<(DHA)
C(20)-H(20)...O(11)#1	0.98	2.44	3.314(4)	147.6
C(14)-H(14)...O(12)#2	0.93	2.57	3.377(4)	145.6
C(20)-H(20)...O(11)#1	0.98	2.44	3.314(4)	147.6
C(16)-H(16)...O(2)	0.93	2.16	2.757(3)	121.3
C(14)-H(14)...O(12)#2	0.93	2.57	3.377(4)	145.6
C(20)-H(20)...O(11)#1	0.98	2.44	3.314(4)	147.6
C(16)-H(16)...O(2)	0.93	2.16	2.757(3)	121.3
C(14)-H(14)...O(12)#2	0.93	2.57	3.377(4)	145.6

Symmetry transformations used to generate equivalent atoms:

#1 x+1,y+1,z #2 -x,y+1/2,-z+3/2

Table S8 .Anisotropic displacement parameters ($Å^2$x 10^3) for **2**. The anisotropic displacement factor exponent takes the form: $-2\ ^2[\ h^2 a^{*2} U^{11} + ... + 2\ h\ k\ a^*\ b^*\ U^{12}\ ]$

	U^{11}	U^{22}	U^{33}	U^{23}	U^{13}	U^{12}
O(3)	62(1)	54(1)	71(1)	-3(1)	-9(1)	2(1)
O(27)	46(1)	59(1)	67(1)	-12(1)	-3(1)	1(1)
O(30)	75(2)	66(1)	85(2)	-11(1)	-7(1)	-12(1)
O(18)	59(1)	63(1)	108(2)	0(1)	-28(1)	7(1)
C(29)	52(2)	55(1)	45(1)	-1(1)	-3(1)	7(1)
C(13)	50(2)	60(2)	72(2)	-8(1)	-12(2)	13(1)
C(26)	49(2)	56(1)	51(2)	4(1)	-8(1)	-4(1)
O(2)	84(2)	68(1)	160(3)	7(2)	-48(2)	1(1)
C(4)	65(2)	56(1)	54(2)	6(1)	1(2)	2(1)
C(25)	49(2)	59(1)	58(2)	0(1)	-5(1)	-2(1)
O(12)	111(2)	89(2)	120(2)	18(2)	-43(2)	-30(2)
C(20)	46(2)	65(2)	57(2)	4(1)	5(2)	1(1)
C(21)	48(2)	60(1)	52(2)	9(1)	-1(1)	-2(1)
O(11)	128(2)	58(1)	99(2)	4(1)	18(2)	-10(1)
C(24)	66(2)	57(1)	54(2)	4(1)	-7(2)	-7(1)
C(22)	50(2)	75(2)	75(2)	8(2)	-2(2)	-7(2)

C(23)	56(2)	71(2)	76(2)	5(2)	-11(2)	-16(2)
C(17)	47(2)	65(2)	55(2)	2(1)	-7(1)	6(1)
C(16)	61(2)	58(2)	63(2)	2(1)	-7(2)	10(1)
C(28)	55(2)	57(1)	48(1)	1(1)	-5(1)	8(1)
C(19)	58(2)	67(2)	79(2)	2(2)	-23(2)	3(2)
C(14)	50(2)	68(2)	78(2)	-14(2)	-11(2)	7(1)
N(10)	104(3)	66(2)	62(2)	13(1)	-4(2)	-17(2)
C(1)	68(2)	62(2)	69(2)	6(2)	-10(2)	1(2)
C(7)	81(2)	56(2)	51(2)	10(1)	0(2)	-9(2)
C(9)	68(2)	58(2)	66(2)	6(1)	-5(2)	10(1)
C(6)	81(2)	55(2)	73(2)	9(2)	1(2)	5(2)
C(8)	69(2)	66(2)	68(2)	8(2)	-11(2)	-3(2)
C(15)	55(2)	56(1)	50(1)	-1(1)	0(1)	1(1)
C(5)	66(2)	65(2)	71(2)	13(2)	-7(2)	7(2)
C(31)	87(3)	76(2)	103(3)	-24(2)	4(3)	-3(2)

7. DFT Optimization Coordinates for Conformational Analysis of 1(I) and 1(II)

Table S9. Cartesian coordinates and energies optimized by B3LYP/6-311G(*d,p*) for 1(I)

Stoichiometry: C16H14O4

Standard Orientation:

Center Number	Atomic Number	Atomic Type	Coordinates (Angstroms) X	Y	Z
1	6	0	-2.453013	-1.028058	1.317958
2	1	0	-2.243920	-1.365500	2.159340
3	6	0	-3.454468	-1.629204	0.590158
4	1	0	-3.901758	-2.368845	0.933550
5	6	0	-3.794194	-1.125914	-0.662687
6	6	0	-3.135433	-0.010743	-1.161632
7	1	0	-3.370861	0.340791	-1.989330
8	6	0	-2.119818	0.578285	-0.413253
9	6	0	-0.913488	2.567860	-0.029058
10	1	0	-1.614193	2.976219	0.503803
11	1	0	-0.451014	3.274604	-0.505766
12	6	0	0.062536	1.866099	0.888450
13	1	0	0.383239	2.501262	1.562406
14	6	0	1.246093	1.221927	0.213390
15	6	0	2.132050	1.667910	-0.757965
16	1	0	2.010604	2.498706	-1.158494
17	6	0	3.199484	0.863124	-1.127728
18	1	0	3.784368	1.148059	-1.791876
19	6	0	3.400955	-0.369061	-0.511361
20	6	0	2.515899	-0.854632	0.450924
21	1	0	2.635577	-1.685767	0.851640
22	6	0	1.446088	-0.031336	0.777326
23	6	0	-0.576151	0.650606	1.601458
24	1	0	-0.867489	0.909804	2.500694
25	6	0	-1.738598	0.074279	0.835894
26	6	0	4.883941	-2.232868	-0.159877
27	1	0	5.662174	-2.637665	-0.549517
28	1	0	4.162139	-2.865772	-0.157026
29	1	0	5.079187	-1.965579	0.741080
30	8	0	-1.506884	1.667441	-0.979062
31	8	0	0.490315	-0.377554	1.697414
32	8	0	4.508152	-1.084859	-0.927474
33	8	0	-4.774386	-1.680658	-1.443109
34	1	0	-5.216922	-2.204927	-0.994643

Table S10. Cartesian coordinates and energies optimized by B3LYP/6-311G(*d,p*) for 1(II)

Stoichiometry: C16H14O4

Standard Orientation:

Center Number	Atomic Number	Atomic Type	Coordinates (Angstroms) X	Y	Z
1	8	0	0.451721	-1.006993	0.233691
2	8	0	5.218220	-0.564242	-0.447624
3	8	0	-5.853413	-0.874617	-0.645239
4	1	0	-6.185040	-0.181297	-0.931100
5	6	0	-2.578760	-1.496142	0.753111
6	1	0	-2.149675	-2.188169	1.202424
7	8	0	-1.983829	1.932594	-0.478271
8	6	0	1.648817	-0.326349	0.174814
9	6	0	1.531844	1.021472	0.471688
10	6	0	-0.485279	-0.100988	0.967462
11	1	0	-0.438186	-0.286896	1.928513
12	6	0	-1.866178	-0.301251	0.491116
13	6	0	-3.905005	0.580091	-0.573039
14	1	0	-4.348747	1.260297	-1.026153
15	6	0	-3.872078	-1.661897	0.367211
16	1	0	-4.301696	-2.470789	0.528496
17	6	0	2.843645	-0.933552	-0.158119
18	1	0	2.902931	-1.841087	-0.352485
19	6	0	3.945579	-0.098942	-0.182488
20	6	0	-0.580827	1.861102	-0.582786
21	1	0	-0.230862	2.746805	-0.767064
22	1	0	-0.347113	1.287961	-1.329641
23	6	0	-2.583097	0.723442	-0.176992
24	6	0	3.876816	1.250320	0.116709
25	1	0	4.642388	1.777959	0.097145
26	6	0	0.071771	1.321023	0.682875
27	1	0	-0.066678	1.921781	1.444664
28	6	0	2.638209	1.808216	0.448997
29	1	0	2.572702	2.713134	0.653300
30	6	0	-4.552985	-0.637438	-0.262868
31	6	0	5.376381	-1.971530	-0.648851
32	1	0	4.989112	-2.446010	0.090407
33	1	0	6.310768	-2.182934	-0.710605
34	1	0	4.935393	-2.230154	-1.461408
